# The long noncoding RNA APR attenuates PPRV infection-induced accumulation of intracellular iron to inhibit membrane lipid peroxidation and viral replication

**DOI:** 10.1128/mbio.00127-25

**Published:** 2025-03-24

**Authors:** Bo Wen, Wenchi Chang, Lulu Yang, Daiyue Lv, Lizhen Wang, Lei Wang, Yanzhao Xu, Jianhe Hu, Ke Ding, Qinghong Xue, Xuefeng Qi, Bo Yang, Jingyu Wang

**Affiliations:** 1 College of Animal Science and Veterinary Medicine, Henan Institute of Science and Technology 177560 , Xinxiang, Henan, China; 2 College of Veterinary Medicine, Northwest A&F University 718173 , Yangling, Shaanxi, China; 3 Key Laboratory of Ruminant Disease Prevention and Control (West), Ministry of Agriculture and Rural Affairs 12654 , Xi'an, Shaanxi, China; 4 China Institute of Veterinary Drug Control 620909 , Beijing, Beijing, China; 5 College of Veterinary Medicine, Shanxi Agricultural University 74600 , Taigu, Shanxi, China; Virginia Polytechnic Institute and State University, Blacksburg, Virginia, USA

**Keywords:** PPRV, iron overload, lipid peroxidation, lncRNA APR, replication

## Abstract

Peste des petits ruminants virus (PPRV) is an important pathogen that has long been a significant threat to small ruminant productivity worldwide. Iron metabolism is vital to the host and the pathogen. However, the mechanism underlying host-PPRV interactions from the perspective of iron metabolism and iron-mediated membrane lipid peroxidation has not been reported thus far. In this study, we identified a novel host long-noncoding RNA (lncRNA), APR, that impairs PPRV infectivity by sponging miR-3955-5p, a negative microRNA (miRNA) that directly targets the gene encoding the ferritin-heavy chain 1 (FTH1) protein. Importantly, we demonstrated that PPRV infection causes aberrant cellular iron accumulation by increasing transferrin receptor (TFRC) expression and that iron accumulation induces reticulophagy and ferroptosis, which benefits PPRV replication. Moreover, PPRV infection enhanced the localization of cellular iron on the endoplasmic reticulum (ER) and caused ER membrane damage by promoting excess lipid peroxidation to induce reticulophagy. Interestingly, APR decreased PPRV infection-induced accumulation of intracellular Fe^2+^ via miR-3955-5p/FTH1 axis and ultimately inhibited reticulophagy and ferroptosis. Additionally, our results indicate that interferon regulatory factor 1 promotes APR transcription by positively regulating APR promoter activity after PPRV infection. Taken together, our findings revealed a new pattern of PPRV-host interactions, involving noncoding RNA regulation, iron metabolism, and iron-related membrane lipid peroxidation, which is critical for understanding the host defense against PPRV infection and the pathogenesis of PPRV.

**IMPORTANCE:**

Many viruses have been demonstrated to engage in iron metabolism to facilitate their replication and pathogenesis. However, the mechanism by which PPRV interacts with host cells from the perspective of iron metabolism, or iron-mediated membrane lipid peroxidation, has not yet been reported. Our data provide the first direct evidence that PPRV infection induces aberrant iron accumulation to promote viral replication and reveal a novel host lncRNA, APR, as a regulator of iron accumulation by promoting FTH1 protein expression. In this study, PPRV infection increased cellular iron accumulation by increasing TFRC expression, and more importantly, iron overload increased viral infectivity as well as promoted ER membrane lipid peroxidation by enhancing the localization of cellular iron on the ER and ultimately induced ferroptosis and reticulophagy. Furthermore, a host factor, the lncRNA APR, was found to decrease cellular iron accumulation by sponging miR-3955-5p, which directly targets the gene encoding the FTH1 protein, thereby attenuating PPRV infection-induced ferroptosis and reticulophagy and inhibiting PPRV infection. Taken together, the results of the present study provide new insight into our understanding of host-PPRV interaction and pathogenesis from the perspective of iron metabolism and reveal potential targets for therapeutics against PPRV infection.

## INTRODUCTION

Peste des petits ruminants (PPR) is an acute, highly contagious and fatal viral disease of sheep and goats with a widespread distribution that causes significant economic losses (1, 2). The disease is clinically characterized by a high fever, mucopurulent oculonasal discharge, diarrhea, stomatitis, and pneumonia symptoms (3). The large impact of the PPR on small ruminant production operations has led the World Organization for Animal Health (WOAH) to list the disease as an important and notifiable animal disease (3). PPRV, which is an enveloped negative-stranded RNA virus belonging to the *Paramyxoviridae* family and the *Morbillivirus* genus, is the causative agent of PPR (4). Like morbilliviruses, PPRV shows lymphatic and epithelial tropism and usually causes severe immune suppression in the host (5–7).

Noncoding RNAs (ncRNAs), accounting for more than 98% of DNA transcripts, are a class of transcripts that exhibit very low or no coding ability and can be divided according to length, into short noncoding RNAs (<200 nt) and long noncoding RNAs (lncRNAs, >200 nt) (8, 9). lncRNAs regulate gene expression at the transcriptional and post-transcriptional levels by interacting with DNA, proteins, or other RNAs in the nucleus or cytoplasm and play vital roles in a wide variety of important biological processes and diseases (10, 11). Notably, lncRNAs participate in the battle between hosts and viruses and play regulatory roles in autophagy and ferroptosis by influencing the post-transcriptional modification and stability of ferroptosis-related proteins (11–15). In our previous study, we identified a system of significantly differentially expressed lncRNAs from PPRV-infected caprine endometrial epithelial cells (EECs) and discovered an interaction between the lncRNA interferon regulatory factor 1 (IRF1)-AS and PPRV infection (16). MicroRNAs (miRNAs), which are 20–25 nt in length, are the short, noncoding RNAs that mainly regulate the degradation or translation inhibition of target genes at the post-transcriptional level by binding to the 3′-untranslated regions (UTRs) (17). An increasing number of studies have investigated the role of miRNAs in viral replication and have indicated that they can inhibit or promote viral infection (18, 19). We have revealed the roles of a system of miRNAs in PPRV replication and pathogenesis (7, 20, 21). Overall, ncRNAs play crucial roles in PPRV infection. Nevertheless, how lncRNAs cooperate with miRNAs to regulate PPRV infection has still not been studied.

Iron, an essential mineral element for almost all living cells and organisms, has been shown to be pivotal for physiological organism growth and the survival of tissues and organs (22). Generally, the basic process of iron metabolism in animals and humans includes iron absorption, iron storage, and iron excretion (22). Transferrin (TF)-bound ferric iron in the serum is recognized by the transferrin receptor (TFRC/TFR1), a TF carrier protein in cell membranes, and then transported into cells (23). Cellular iron is stored mainly by ferritin, a ubiquitous iron-binding protein consisting of a mixture of 24 subunits comprised of the ferritin heavy chain (FTH1) and ferritin light chain (FTL) (24). Iron metabolism balance is critical for normal body functions; however, dysregulated iron homeostasis (mainly iron overload) is involved in the development of different diseases (25, 26). Although iron homeostasis has been reported to affect the proliferation of several viruses (27–29), the role of intracellular iron in the replication and pathogenesis of PPRV is still unclear.

Iron participates in the peroxidation of membrane-bound, polyunsaturated fatty acids (PUFA)-containing lipids by facilitating the Fenton reaction, which propagates lipid peroxidation (30), and is associated with iron-dependent enzymes that initiate the formation of lipid hydroperoxide substrates in the Fenton reaction (31). When the peroxidation of membrane-bound lipids reaches lethal levels via intracellular iron overload, in other words, when lipid hydroperoxides accumulate in cellular membranes, ferroptosis, a newly recognized form of regulated cell death, is triggered (32). As an intracellular organelle enriched with membrane-bound lipids, the endoplasmic reticulum (ER) is an essential site of lipid peroxidation in ferroptosis (33). Lipid hydroperoxides produced by lipid peroxidation, especially those of membrane-bound lipids, alter the assembly, structure, and dynamics of biological membranes, resulting in membrane damage, membrane thinning and increased curvature (34). Lipid peroxidation, which is increased by iron overload, is believed to lead to a breakdown of ER membranes (35, 36), and excess iron or accelerated lipid peroxidation can even elicit ER stress (ERS) (37, 38). In addition, ERS can be relieved by autophagy, and parts of the ER can be degraded by ER-specific autophagy, a process referred to as reticulophagy (39).

Ferroptosis and its contribution to the replication and pathogenesis of several viruses, including Newcastle disease virus (NDV), bovine viral diarrhea virus (BVDV) influenza A virus (IAV), Epstein-Barr virus (EBV), and human immunodeficiency virus (HIV) (40–45) have been investigated; however, the relationships between PPRV and the peroxidation of membrane-bound lipids and ferroptosis, have not been investigated to date. In addition, our work and that of others have indicated that PPRV infection induces autophagy, which is mediated by ERS (46–48), However, whether PPRV infection-induced ERS is mediated by the peroxidation of ER membrane-bound lipids and leads to reticulophagy needs to be further investigated. In this study, we revealed the relationship between PPRV infection and iron metabolism or iron-mediated membrane-bound lipid peroxidation and elucidated the underlying mechanism by which the host noncoding RNAs regulate iron metabolism, which contributes to our understanding of how PPRV interacts with host cells in a new perspective.

## RESULTS

### The lncRNA APR is aberrantly upregulated by PPRV infection and inhibits PPRV replication

**Fig 1:**
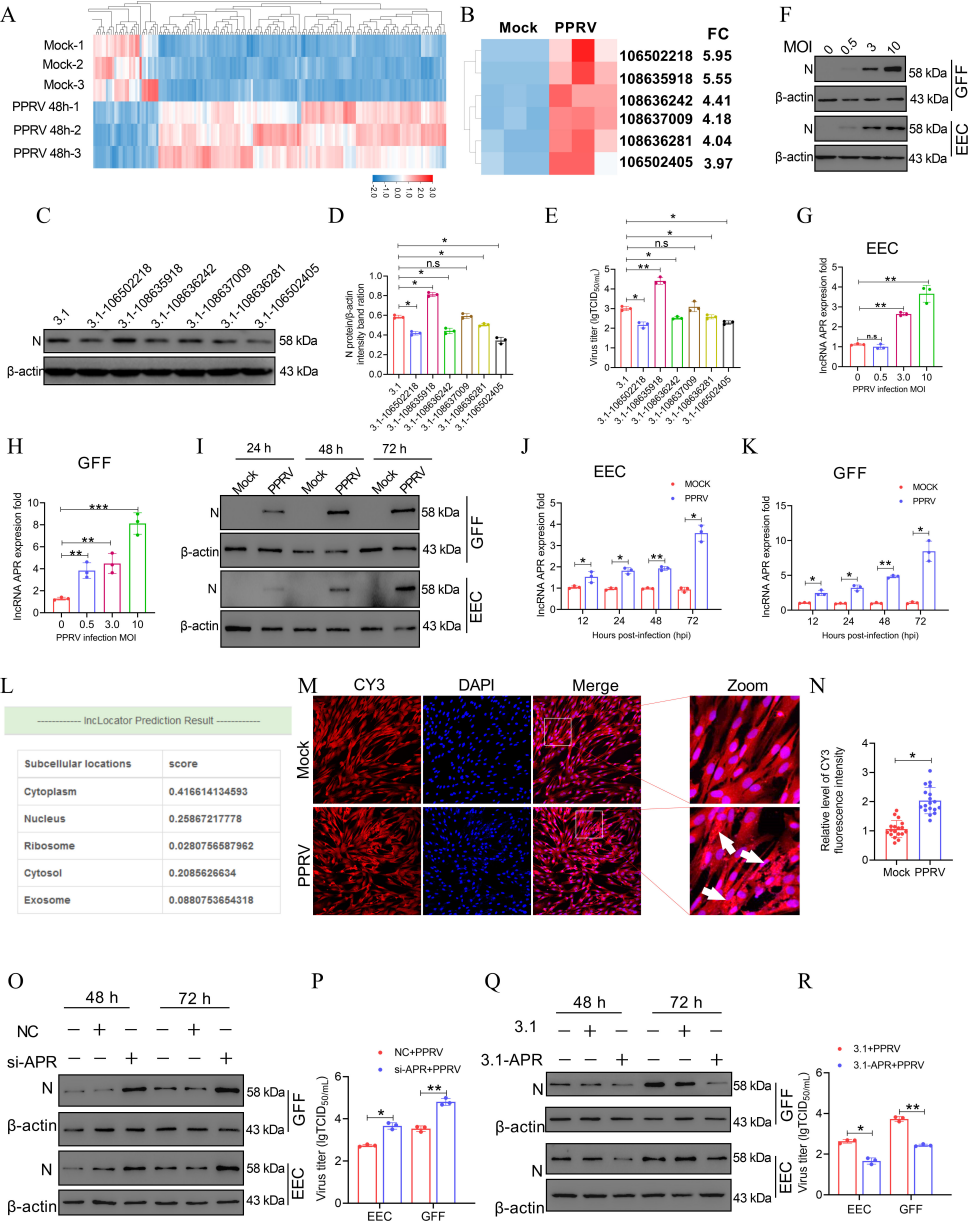
PPRV infection promoted the aberrant high expression of long, noncoding RNA APR that inhibits viral replication. (A) A total of 191 differentially expressed lncRNAs were identified by RNA-seq analysis in PPRV-infected EECs compared with mock-infected cells. These lncRNAs were clustered and shown by heatmap. (B) Six of the differentially expressed lncRNAs were selected and clustered in a heatmap. (C–E) The indicated upregulated lncRNAs were selected and overexpressed in EECs by transfecting with indicated plasmids. Then the cells were infected with PPRV at a MOI of 3. 48 h later, (C) Western blot and (E) TCID_50_ assays were performed to determine the viral replication and progeny. (D) The relative quantification of N protein levels compared with β-actin protein levels was determined by densitometry. (F) EECs or GFFs were infected with PPRV at different MOIs for 48 h, and then, the N protein expression was measured by western blot. (G) EECs or (H) GFFs were infected with PPRV at different MOIs for 48 h, and then, the APR expression was measured using the qRT-PCR assay. (I) EECs or GFFs were infected with PPRV at an MOI of 3 for the indicated times, and then, the N protein expression was measured by western blot. (J) EECs or (K) GFFs were infected with PPRV at an MOI of 3 for the indicated times, and then, the APR expression was measured by qRT-PCR assay. (L) The prediction the subcellular localization of APR by online website. (M and N) EECs were infected or mocked infected with PPRV at an MOI of 3 for 48 h, (M) then, the localization of APR in EECs was examined by RNA-FISH. (N) Quantitative analysis of CY3. (O and P) EECs and GFFs were transfected with nonspecific control siRNA (NC) or siRNA against APR (si-APR) for 24 h and then infected with PPRV at an MOI of 3 for 48 h and 72 h. Then, the cells were subjected to (O) western blot, and (P) the virus titers in the supernatants were measured by TCID_50_ assay. (Q and R) EECs and GFFs were transfected with pcDNA3.1 or pcDNA 3.1-APR for 24 h and then infected with PPRV at an MOI of 3 for 48 h and 72 h. Then, (Q) the cells were subjected to western blot, and the (R) virus titers in the supernatants were measured by TCID_50_ assay. The data represent the mean ± SD of three independent experiments. *P* values were calculated using Student’s *t* test. An asterisk indicates a comparison with the indicated control. *P < 0.05; **P < 0.01; ***P < 0.001; n.s., not significant.

lncRNAs have been proven to participate in the battle between the host and the virus (9). We previously reported that a panel of lncRNAs was markedly upregulated after infection with the PPRV vaccine strain N75-1 in EECs (16) (Fig. 1A; Table S1). Given that lncRNAs that accumulate in response to viral infection may participate in cellular responses to PPRV, we cloned six of the most highly upregulated lncRNAs (Fig. 1B; Table S1) and overexpressed them in EECs (Fig. S1A). These cells were then infected with PPRV at a multiplicity of infection (MOI) of 3. We observed that the six lncRNAs expressed, respectively, in EECs showed different effects on PPRV replication, and importantly, one of these lncRNAs—loc106502218—was found to inhibit PPRV infection (Fig. 1C through E). Although loc106502405 also had an inhibitory effect on virus replication similar to that of loc10652218, loc106502218 showed an upregulated trend in most fold changes during PPRV infection. Therefore, loc106502218 was selected for further study. To investigate the relationship between PPRV infection and loc106502218 activation, we determined the virus levels and loc10652218 transcription levels in EECs and in goat fetal fibroblasts cells (GFFs) infected with PPRV via reverse transcription‒quantitative PCR (qRT‒PCR). The results indicated that loc106502218 was a PPRV infection-responsive gene in host cells, and its induction was accompanied by increased virus levels either in a dose-dependent manner (Fig. 1F through H) or in an infection time-dependent manner (Fig. 1I through K). The exact transcript length (1,463 nt) of loc10652218 was subsequently confirmed by rapid amplification of cDNA ends (RACE) (Table S2). Since loc106502218 was found to be associated with PPRV replication, we named it the lncRNA associated with PPRV replication (APR). In addition, we predicted of the subcellular localization of APR via http://www.csbio.sjtu.edu.cn/bioinf/lncLocator/, which revealed that APR was located in both the cytoplasm and nucleus (Fig. 1L). RNA-fluorescence *in situ* hybridization (RNA-FISH) was also performed with mock- or PPRV-infected EECs, and the results revealed that APR was distributed in both the nucleus and the cytoplasm (Fig. 1M and N). Importantly, RNA-FISH revealed that when compared with the level in the mock-infected groups, PPRV infection increased the RNA level of loc106502218 in the cytoplasm, but not in the nucleus.

To evaluate the influence of APR on PPRV proliferation, we knocked down APR in EECs or GFFs via RNA interference, and qRT‒PCR was performed to determine the APR transcript level. The cells were subsequently infected with PPRV for the indicated times. The cell lysates were harvested for immunoblotting, and the supernatants were collected for 50% tissue culture infective dose (TCID_50_) assays. Our results indicated that treatment with siRNA specific for APR (si-APR) reduced the amount of APR in EECs (Fig. S1B) and GFFs (Fig. S1C). Both the protein level of N and the viral titer were higher in the APR-knockdown cells than in the control cells (Fig. 1O and P). Next, we transfected EECs and GFFs with a pcDNA3.1 plasmid expressing APR (pcDNA 3.1-APR) or an empty vector (pcDNA 3.1), and the cells were subsequently infected with PPRV. qRT‒PCR was performed to determine the efficiency of overexpression (Fig. S1D and E). PPRV replication was analyzed as described above. Our results revealed that overexpressing APR reduced the level of the PPRV N protein (Fig. 1Q) and the virus titer (Fig. 1R) compared with those in mock-overexpressing cells.

Taken together, these results demonstrated that the lncRNA that we identified, APR, is an aberrantly highly expressed ion level after PPRV infection and that, in turn, APR inhibits PPRV replication.

### APR plays a negative role in PPRV replication by targeting the miR-3955-5p/FTH1 axis

**Fig 2:**
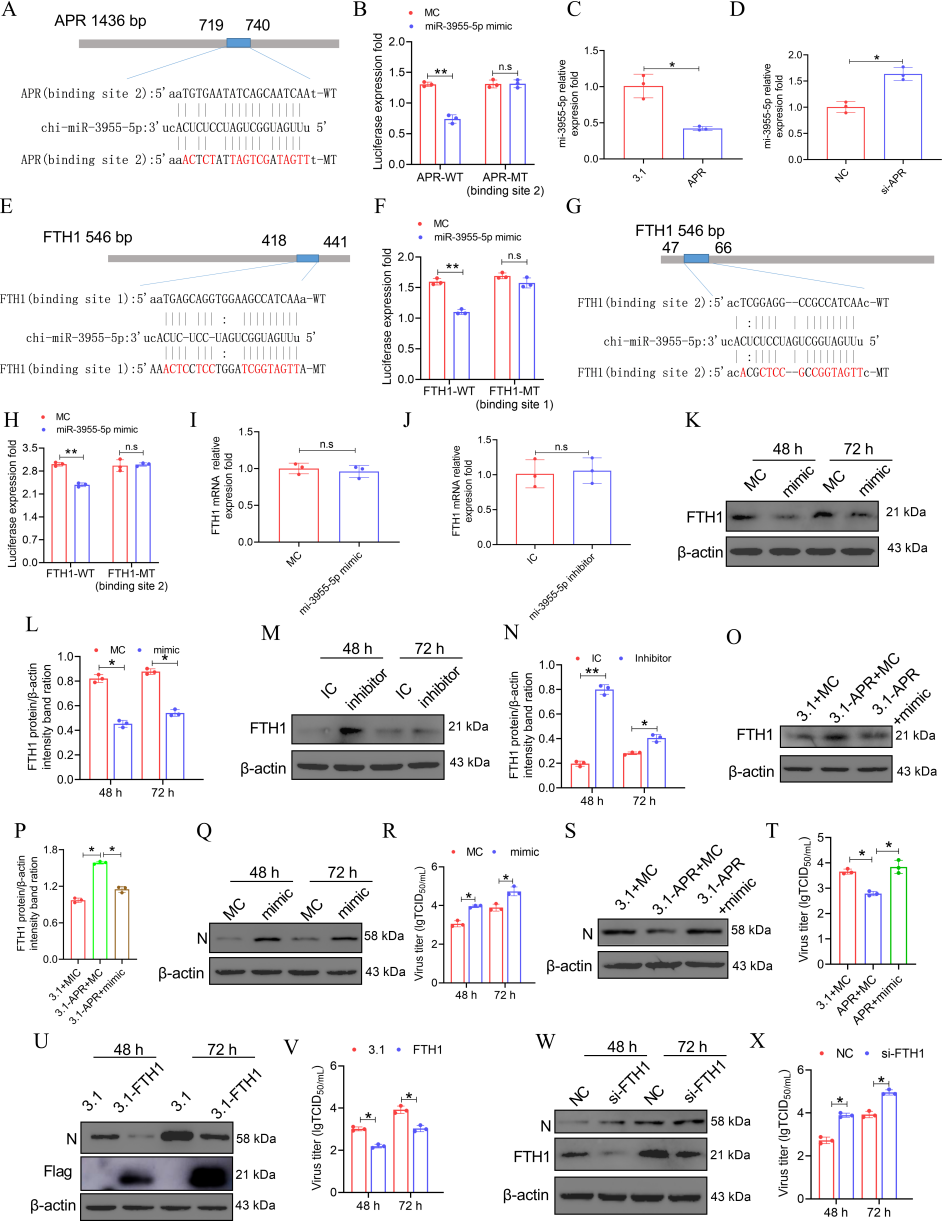
APR inhibits PPRV replication by targeting the miR-3955-5p/FTH1 axis. (A). Sequence alignment of miR-3955-5p and its binding site 2 in APR, as predicated by TargetScan algorithm software. (B) Dual-luciferase assay of HEK293T cells co-transfected with pmirGLO containing the putative miR-3955-5p binding site 2, or pmirGLO containing the mutated binding sites, together with synthetic mature miR-3955-5p (miR-3955-5p mimic) or mimic control (MC). (C and D) The expressions of miR-3955-5p (C) in the APR overexpression EECs and (D) in the APR knockdown EECs were measured by qRT-PCR. (E and G) Sequence alignment of miR-3955-5p and (E) its binding site 1 and (G) binding site 2 in FTH1, as predicated by TargetScan algorithm software. (F and H) Dual-luciferase assay of HEK293T cells co-transfected with pmirGLO containing (F) the putative miR-3955-5p binding site 1 and (H) binding site 2, or pmirGLO containing the mutated binding sites, together with miR-3955-5p mimic or (MC). (I and J) The expressions of FTH1 mRNA (I) in the miR-3955-5p overexpression EECs or (J) in the miR-3955-5p knockdown EECs were measured by qRT-PCR. (K–N) EECs were transfected with (K and L) miR-3955-5p mimic or (M and N) miR-3955-5p inhibitor and the indicated control (control mimic (MC) or control inhibitor (IC), 24 h later, the cells were infected with PPRV at an MOI of 3 for indicated time point, then (K and M) the protein expression of FTH1 was measured by western blotting. (L and N) The relative quantification of FTH1 protein levels compared with β-actin protein levels was determined by densitometry. (O, P, S, and T) EECs were cotransfected with pcDNA3.1 empty vector or pcDNA3.1-APR and control mimic (MC) or miR-3955-5p mimic for 24 h, and then infected with PPRV at an MOI of 3 for 48 h. Then, the cells were subjected to western blot for the analysis of the expression of (O and P) FTH1 and (S) N protein, and (T) the virus titers in the supernatants were measured by TCID_50_ assay. (Q and R) EECs were transfected with MC or miR-3955-5p mimic, and 24 h later, the cells were infected with PPRV at an MOI of 3 for the indicated time point. Then, the cells were subjected to (Q) western blot, and (R) the virus titers in the supernatants were measured by TCID_50_ assay. (U and V) EECs were transfected with pcDNA3.1 or pcDNA 3.1-FTH1 for 24 h and then infected with PPRV at an MOI of 3, 48, and 72 h later; (U) the N protein expression was tested by western blot, and (V) the virus titers in the supernatants were measured by TCID_50_ assay. (W and X) EECs were transfected with nonspecific control siRNA (NC) or siRNA against FTH1 (si-FTH1), for 24 h and then infected with PPRV at an MOI of 3, 48, and 72 h later; (W) the N protein expression was tested by western blot, and (X) the virus titers in the supernatants were measured by TCID_50_ assay. β-Actin was used as a loading control in western blot analysis. The data represent the mean ± SD of three independent experiments. *P* values were calculated using Student’s *t* test. An asterisk indicates a comparison with the indicated control. *P < 0.05; **P < 0.01; n.s., not significant.

Interactions between lncRNAs and miRNAs, which constitute an important class of noncoding RNAs in eukaryotes, provide an additional layer of control in gene regulation (49). To reveal the underlying mechanisms of the negative role of APR in PPRV replication, three miRNAs, chi-miR-324-3p, chi-miR-106b-3p, and chi-miR-3955-5p, which putatively bind to APR, were identified via miRanda and RNAhybrid. Furthermore, we predicted the possible binding sites for chi-miR-324-3p, chi-miR-106b-3p, and chi-miR-3955-5p with APR. These sites were from nucleotides 422–446 for miR-3955-5p-binding site 1 (Fig. S2A), from nucleotides 719–740 for miR-3955-5p-binding site 2 (Fig. 2A), from nucleotides 363–385 for miR-324-3p (Fig. S2E), and from nucleotides 1178–1197 for miR-106-3p (Fig. S2C). A dual-luciferase reporter assay showed that cotransfection of the pmirGLO-APR-WT luciferase reporter vector, which carries the miR-3955-5p-binding site 2 (Fig. 2B) but not the miR-3955-5p-binding site 1 (Fig. S2B), but not the pmirGLO-APR-MT luciferase reporter vector, which carries miR-3955-5p mimics, markedly reduced APR-regulated luciferase activity in HEK293T cells. However, cotransfection of pmirGLO-APR-WT (carrying binding sites for miR-324-3p or miR-106b-3p) with miR-324-3p or miR-106b-3p mimics did not reduce APR-regulated luciferase activity in HEK293T cells (Fig. S2D and F). Furthermore, APR overexpression or knockdown decreased and increased the level of miR-3955-5p, respectively (Fig. 2C and D). These results demonstrated that APR sponges miR-3955-5p.

Although miRNAs can regulate various biological processes through perfect or partial base pairing in the 3′-UTR regions of target mRNAs (50), interestingly, we predicted that miRNA-3955-5p bound to the FTH1 coding DNA sequence (CDS), which includes two-miRNA binding sites (Fig. 2E and G). Importantly, a dual-luciferase reporter assay revealed that the luciferase activity of the pmirGLO-FTH1-WT (carrying binding site one and binding site two for miR-3955-5p) reporter vector was significantly decreased in the presence of the miRNA-3955-5p mimic (Fig. 2F and H). Additionally, we transfected EECs with a miRNA-3955-5p mimic or inhibitor to investigate whether miRNA-3955-5p affected FTH1 expression. Although the results indicated that transfection of the miRNA-3955-5p mimic or miRNA-3955-5p inhibitor did not affect FTH1 mRNA transcription (Fig. 2I and J), the protein expression of FTH1 was decreased by the miRNA-3955-5p mimic (Fig. 2K and L) and increased by the miRNA-3955-5p inhibitor in EECs (Fig. 2M and N).To determine whether APR modulated FTH1 expression by adsorbing miRNA-3955-5p, we cotransfected cells with a pcDNA3.1 empty vector or pcDNA3.1-APR and miRNA control (MC). In the rescue group, pcDNA3.1-APR and the miRNA-3955-5p mimic were cotransfected into EECs. The cells were harvested to evaluate FTH1 protein expression. Our results indicated that FTH1 protein upregulation induced by APR transfection was reversed by the presence of the miRNA-3955-5p mimic (Fig. 2O and P). Together, these results demonstrated that APR regulates FTH1 protein expression by sponging miRNA-3955-5p in EECs.

Additionally, we assessed whether APR affects PPRV infection via the miR-3955-5p/FTH1 axis. PPRV infection assays were performed in EECs pretransfected with miR-3955-5p mimics or the respective controls. PPRV infection was examined by Western blot and TCID_50_ assays. Our data revealed that the miR-3955-5p mimic increased the level of the PPRV N protein (Fig. 2Q) and the virus titer (Fig. 2R). Moreover, miR-3955-5p reversed the inhibitory effect of APR on PPRV infection (Fig. 2S and T). FTH1 is the final molecule in the miR-3955-5p/FTH1 axis, and the level of the PPRV N protein (Fig. 2U) and the virus titer (Fig. 2V)were both decreased in FTH1-overexpressing cells but increased in FTH1-knockdown cells (Fig. 2W and X) during PPRV infection. Collectively, our data clearly revealed that APR can inhibit PPRV infection via the miR-3955-5p/FTH1 axis.

### lncRNA APR decreases intracellular Fe^2+^ accumulation caused by PPRV infection via the miR-3955-5p/FTH1 axis

**Fig 3:**
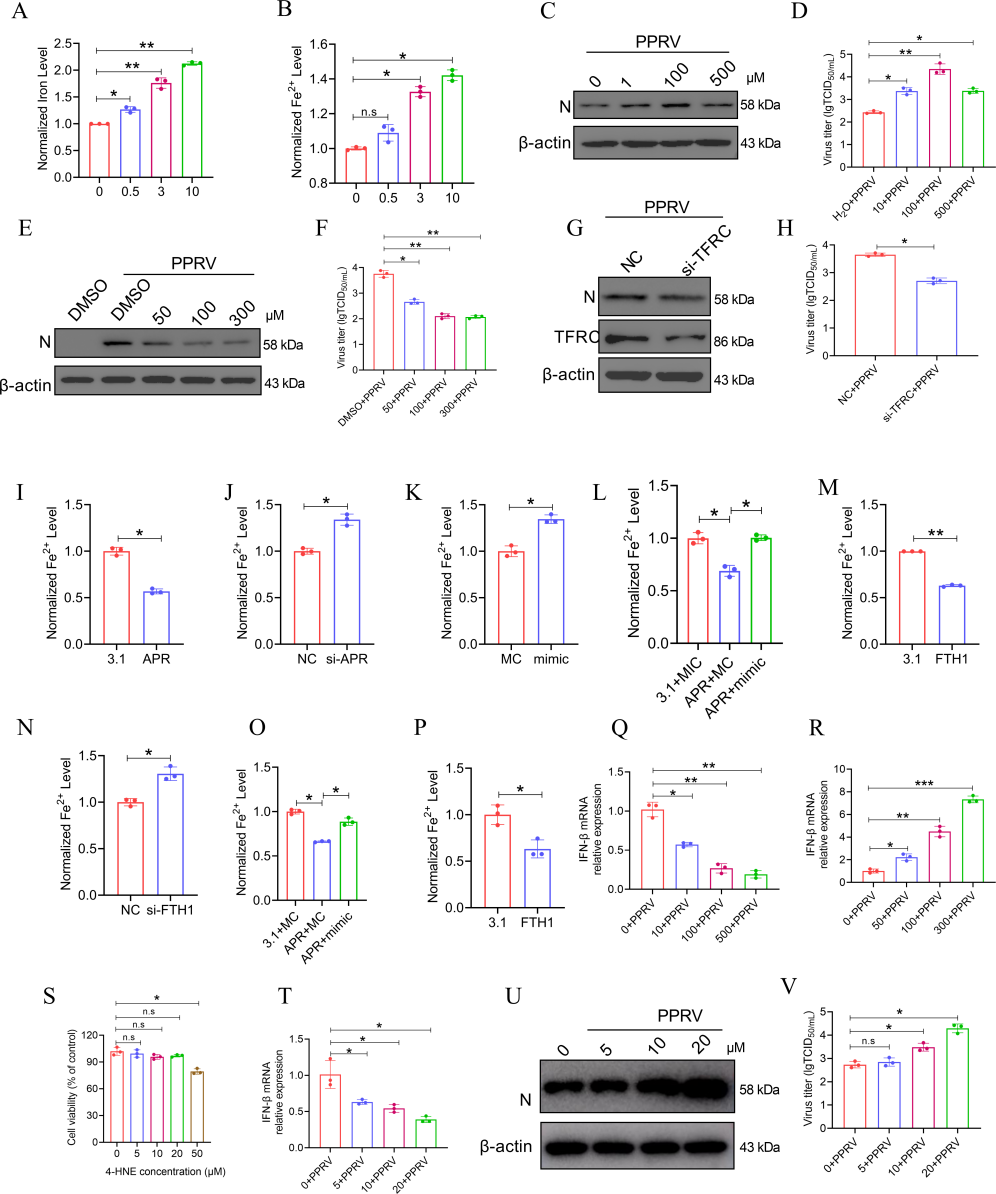
lncRNA APR decreases intracellular Fe^2+^ accumulation caused by PPRV infection via the miR-3955-5p/FTH1 axis to impair viral replication. (A and B) EECs were infected with PPRV at different MOIs (MOI = 0, 0.5, 3, 10) for 48 h and then (A) level of total iron and (B) level of Fe^2+^ were detected. EECs were pre-treated (C and D) with different concentrations of FAC or mocked treated (H_2_O) for 2 h, or (E and F) with different concentrations of DFOM or mocked treated (DMSO) for 2 h. Then, the cells were infected with PPRV at an MOI of 3 for 48 h, and (C and E) Western blot and (D and F) TCID_50_ assays were performed to determine the viral replication and progeny. (G and H) EECs were transfected with NC and siRNA against TFRC (si-TFRC) for 24 h. Then, the cells were infected with PPRV at an MOI of 3 for 48 h, and (G) Western blot and (H) TCID_50_ assays were performed to determine the viral replication and progeny. (I and J) EECs were transfected with (I) pcDNA 3.1-APR or (J) si-APR for 24 h and then infected with PPRV at an MOI of 3, 48 h later, levels of ferrous iron were detected. (K) EECs were transfected with MC or miR-3955-5p mimic for 24 h and then infected with PPRV at an MOI of 3 for 48 h, and then level of ferrous iron was detected. (L) EECs were cotransfected with pcDNA3.1 empty vector or pcDNA3.1-APR and MC or miR-3955-5p mimic for 24 h and then the cells were infected with PPRV at an MOI of 3 for 48 h. Then, level of ferrous iron was detected. EECs were transfected with (M) pcDNA3.1-FTH1 or (N) si-FTH1 for 24 h and then infected with PPRV at an MOI of 3, 48 h and 72 h later, level of ferrous iron was detected. (O) EECs were cotransfected with pcDNA3.1 or pcDNA3.1-APR and MC or miR-3955-5p mimic for 24 h and then the cells were treated with FAC (100 µM), 48 h later. The level of ferrous iron was detected. (P) EECs were transfected with pcDNA3.1 or pcDNA3.1-FTH1 for 24 h and then the cells were treated with FAC (100 µM) for 48 h. Then the level of ferrous iron was detected. EECs were pre-treated with different concentrations of FAC (Q) or with different concentrations of DFOM (R) for 2 h. Then, the cells were infected with PPRV at an MOI of 3 for 48 h, and IFN-β expression was measured by qRT-PCR assay. (S) The cell viability of EECs was measured by CCK-8 after treatment with different concentrations of 4-HNE for 48 h. (U-V) EECs were pre-treated with different concentrations of 4-HNE for 2 h. Then, the cells were infected with PPRV at an MOI of 3 for 48 h. Then, (T) IFN-β expression was measured by qRT-PCR assay, (U) N protein expression was measured by Western blotting and (V) TCID_50_ assays were performed to determine the viral progeny. The data represent the mean ± SD of three independent experiments. *P* values were calculated using Student’s *t* test. An asterisk indicates a comparison with the indicated control. *P < 0.05; **P < 0.01.

FTH1 is a subunit of ferritin, which stores excess cellular iron (51). Naturally, we speculated that APR regulates cellular Fe^2+^ levels, and modulates cellular iron homeostasis, via the miR-3955-5p/FTH1 axis to inhibit PPRV replication. Before we test our hypothesis, the relationship between the cellular iron concentration and PPRV infection was studied. The data revealed that the intracellular concentrations of total iron (Fe) (Fig. 3A), and ferrous iron (Fe^2+^) (Fig. 3B) were increased in EECs infected with PPRV in a dose-dependent manner, which indicated that PPRV infection induces iron aberrant accumulation, in other words, iron overload, in host cells.

To investigate whether PPRV infection-induced iron overload affects PPRV replication, ferric ammonium citrate (FAC) was incubated with EECs, and 2 h later, the cells were infected with PPRV. The infected cells were subsequently cultured in a medium supplemented with FAC. After 48 h of PPRV infection, the cells were collected and subjected to western blotting, and the supernatants were collected for TCID_50_ analysis to determine viral propagation. Our results indicated that FAC treatment significantly decreased cell viability (Fig. S3A) and increased the total iron (Fe) (Fig. S3B) and Fe^2+^ levels in a dose-dependent manner (Fig. S3C) and the PPRV N protein levels (Fig. 3C) and virus titers (Fig. 3D) compared with those in control cells. Moreover, EECs were treated with iron chelator deferoxamine (DFOM), before infection with PPRV and maintained in DFOM-supplemented media for 48 hpi to prevent PPRV-induced iron overload. DFOM at 100 µM did not cause significant cell death (Fig. S3D). Therefore, 100 µM was used as the optimum concentration for DFOM in other experiments. The results indicated that the increase in intracellular Fe^2+^ caused by PPRV infection was abolished in the DFOM-treated cells (Fig. S3E). Western blot and TCID_50_ analyses demonstrated that DFOM impaired viral replication and progeny formation (Fig. 3E and F). To further determine the role of iron in PPRV replication, EECs were transfected with a siRNA targeting TFRC, a carrier protein that is critical for transporting iron into cells, followed by PPRV infection for the indicated times. Our data revealed that treatment of the siRNA-transfected cells significantly decreased the Fe^2+^ level in the PPRV-infected EECs (Fig. S3F) and inhibited PPRV replication (Fig. 3G and H). Collectively, our results clearly revealed that PPRV infection induces iron overload to facilitate viral replication.

Considering that APR increases FTH1 protein expression by sponging miRNA-3955-5p, we explored whether APR plays a role in PPRV infection-induced iron overload. Our results indicated that APR overexpression decreased the intracellular concentration of Fe^2+^ in PPRV-infected EECs (Fig. 3I). However, APR knockdown significantly increased the intracellular concentration of Fe^2+^ in PPRV-infected EECs (Fig. 3J). Next, we explored whether APR regulates cellular Fe^2+^ levels and modulates cellular iron homeostasis, via the miR-3955-5p/FTH1 axis. The miR-3955-5p mimic increased the intracellular concentration of Fe^2+^ in PPRV-infected EECs (Fig. 3K) and reversed the inhibition of Fe^2+^ accumulation (Fig. 3L) resulting from APR overexpression in PPRV-infected cells. Additionally, EECs overexpressing FTH1 after transfection with a pcDNA31-FTH1 plasmid before infection with PPRV exhibited tolerance to iron overload (Fig. 3M), and the opposite results were found when FTH1 was downregulated by siRNA-induced knockdown (Fig. 3N). What’s more, we treated EECs with FAC (100 µM) after cotransfecting them with a pcDNA3.1 empty vector or with pcDNA3.1-APR and miRNA-3955-5p MC to establish the experimental group or after transfecting them with pcDNA3.1-APR and a miRNA-3955-5p mimic to establish the rescue group. The results indicated that the miRNA-3955-5p mimic abolished the APR-mediated inhibition of FAC-induced iron accumulation (Fig. 3O) in EECs. EECs overexpressing FTH1 in the FAC treatment environment also exhibited tolerance to iron overload (Fig. 3P). Recently, it has been reported that lipid peroxidation mediated by iron overload impairs antiviral response by producing excess lipid peroxides to regulate virus replication. We also found that FAC could promote IFN-β (Fig. 3Q) and DFOM impairs IFN-β expression (Fig. 3R). Importantly, 4-HNE, a lipid peroxide, could block IFN-β expression (Fig. 3T) and enhances virus repliction (Fig. 3U and V).

Thus, we concluded that APR reduces the cellular iron level through the miR-3955-5p/FTH1 axis and ultimately attenuates PPRV infection.

### PPRV infection-induced iron overload triggers ferroptosis and reticulophagy to promote PPRV replication

**Fig 4:**
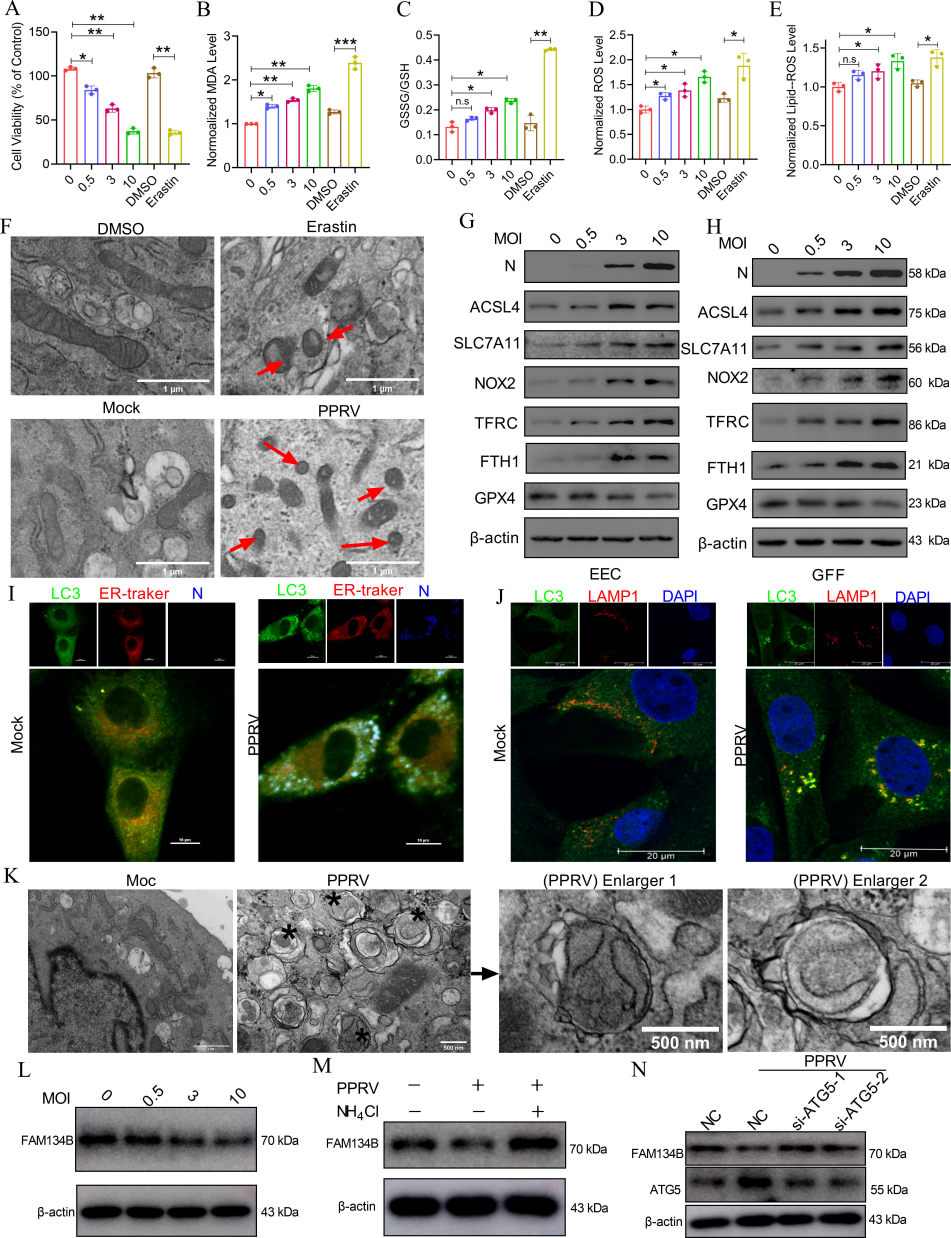
PPRV infection-induced iron overload triggers ferroptosis and reticulophagy to promote PPRV replication. (A–E) EECs were infected with PPRV at different MOIs (MOI = 0, 0.5, 3, 10) for 48 h, and then, the level of (A) MDA, (B) the ratio of GSSG/GSH, (C) level of ROS, (D) level of lipid ROS, and (E) cell viability were detected. (F) Transmission electron microscopy was performed to evaluate the change of mitochondrial ultrastructure in EECs after being treated with erastin (20 µm) or infected by PPRV (MOI = 3) for 48 h. Scale bar: 1 µm. (G) EEC sand (H) GFFs were infected with PPRV (MOI = 3) for 48 h and then subjected to western blot for the analysis of the expression level of ferroptosis-related proteins ACSL4, SLC7A11, NOX2, TFRC, FTH1, and GPX4. (I) EECs were mock-infected or infected with PPRV (MOI = 3) for 48 h, and then, reticulophagy was determined by assessing the colocalization between LC3-positive autophagosomes (green) and ER Tracker labeled endoplasmic reticulum (red) PPRV N protein was labeled (blue). (J) EECs were mock-infected or infected with PPRV (MOI = 3) for 48 h. Then, the cells were fixed and processed for indirect immunofluorescence using antibodies against LC3 and LAMP1 proteins. (K) EECs were mock-infected or infected with PPRV (MOI = 3) for 48 h, and then, reticulophagy was determined by transmission electron microscopy (TEM) analysis. Asterisks point to autophagosomes that include fragments of damaged ER. (L) EECs were infected with PPRV (MOI = 3) for 48 h, and then subjected to western blot for the analysis of the expression level of FAM134B. (M) EECs with or without infection were treated with NH4Cl, which blocks the fusion of autophagosomes and lysosomes, and then, the expression of FAM134B was tested by western blot. (N) EECs were transfected with nonspecific control NC and siRNA against ATG5 (si-ATG5-1/2) for 24 h. Then, the cells were infected PPRV at an MOI of 3 for 48 h, and western blot was performed to test expression of indicated proteins. (O and P) EECs and GFFs were pre-treated with erastin for 2 h and then infected with PPRV (MOI = 3) for 48 and 72 h. Subsequently, (O) western blot, and (P) TCID_50_ assays were performed to determine the viral replication and progeny. (Q and R) EECs and GFFs were pre-treated with ferrostatin-1 (50 or 5 µm) for 2 h and then infected with PPRV (MOI = 3) for 48 h. Subsequently, western blot (Q) and TCID_50_ (R) assays were performed to determine the viral replication and progeny. (S and T) EECs were transfected with nonspecific control NC and siRNA against ATG5 (si-ATG5-1/2) for 24 h. Then, the cells were infected PPRV at an MOI of 3 for 48 h and maintained in culture containing FAC (100 µM) for 48 h, then (S) western blot and (T) TCID_50_ assays were performed to determine the viral replication and progeny. The cell nuclei were counterstained with DAPI (except Fig. 4I). β-Actin was used as a loading control in Western blot analysis. The data represent the mean ± SD of three independent experiments. *P* values were calculated using Student’s *t* test. An asterisk indicates a comparison with the indicated control. *P < 0.05; **P < 0.01.

Iron facilitates the peroxidation of PUFA-containing membrane-localized lipids via the Fenton reaction. When the peroxidation reaches lethal levels via intracellular iron overload, ferroptosis, a newly recognized form of regulated cell death, is triggered (32). Considering that ER is enriched with membrane-bound lipids, iron overload may lead to the breakdown of ER membranes, contributing to ERS and reticulophagy, by accelerating lipid peroxidation in the ER. Therefore, we first determined the relationships between PPRV infection, ferroptosis, and reticulophagy. The data revealed that the intracellular concentrations of total iron (Fe) (Fig. 3A), ferrous iron (Fe^2+^) (Fig. 3B), cell viability (Fig. 4A), malondialdehyde (MDA) (Fig. 4B), the glutathione disulfide (GSSG) to glutathione (GSH) ratio (Fig. 4C), reactive oxygen species (ROS) (Fig. 4D), and lipid ROS (Fig. 4E) were increased in EECs infected with PPRV in a dose-dependent manner. We also treated EECs with erastin, a drug that stimulates ferroptosis, to generate a positive experimental group. For erastin treatment, the cell viability remains at 50% (30 μM) and 60% (20 μM) approximately, and therefore, 20 µM was used as the optimum concentration. It was found that compared with dimethyl sulfoxide (DMSO), this drug caused results similar to those of PPRV infection (Fig. 4A through E). Morphologically, mitochondria in not only the erastin-treated but also the PPRV-infected EECs increasingly shrank with the increasing membrane density (Fig. 4F). Moreover, EECs were treated with the ferrostatin-1 (an inhibitor of ferroptosis) before infection with PPRV and cultured for 48 hpi. It was found that ferrostatin-1 does not influence the cell viability at 5 µM and 50 µM (Fig. S4Q). Therefore, PPRV infection assays were performed with cells treated with ferrostatin-1 at 50 µM. Our results demonstrated that the increases in lipid ROS levels (Fig. S4A), ROS (Fig. S4B), intracellular Fe^2+^ (Fig. S4C), MDA (Fig. S4D), and cell viability (Fig. S4E) caused by PPRV infection were abolished in the ferrostatin-1-treated cells. To further evaluate whether PPRV infection triggers ferroptosis, ferroptosis-related protein levels were measured by western blotting. The results indicated that PPRV infection markedly increased the protein levels of ACSL4, SLC7A11, NOX2, TRFC, and FTH1 but decreased the protein expression levels of GPX4 in EECs (Fig. 4G) and in GFFs (Fig. 4H).

We subsequently investigated whether PPRV infection triggers reticulophagy. Double immunofluorescence staining revealed that PPRV infection increased the colocalization of LC3-II puncta with the ER (Fig. 4I) and with LAMP1 (Fig. 4J) (lysosome marker) in EECs. In addition, cells with or without infection were treated with NH_4_Cl (Fig. S4F and G), which blocks the fusion of autophagosomes and lysosomes, and then, the conversion of LC3-I to LC3-II was assessed. NH_4_Cl promoted the conversion of LC3-I to LC3-II not only in mock-infected cells but also in PPRV-infected cells. The conversion of LC3-I to LC3-II in PPRV-infected cells was more obvious than that in mock-infected cells. Importantly, a transmission electron microscopy (TEM) analysis revealed that in contrast with those in mock-infected cells, some autophagosomes contained parts of the ER whorls in PPRV-infected cells (Fig. 4K). FAM134B is located specially on ER and has identified as a primary ER-phagy receptor (52). FAM134B was found to be degraded in PPRV-infected cells in a dose-dependent manner (Fig. 4L). Additionally, we also found that FAM134B was degraded by the autophagic pathway (Fig. 4M). To investigate whether ATG5 is a regulatory protein specific to reticulophagy, the ATG5 was interfered in PPRV-infected EECs, then the expression of FAMB134B was tested. When ATG was downregulated by specific siRNA, the downregulation of FAMB134B induced by PPRV was rescued (Fig. 4N). The data demonstrated that PPRV infection induces reticulophagy.

To determine whether ferroptosis induced by PPRV infection is mediated by iron overload in EECs, we used FAC to simulate iron overload conditions in EECs. FAC increased the MDA (Fig. S4H) and lipid ROS levels (Fig. S4I). To further determine the effect of iron on PPRV infection-induced ferroptosis, EECs were treated with the DFOM before infection with PPRV and cultured for 48 hpi. Our results demonstrated that the increases in MDA (Fig. S4J) and lipid ROS levels (Fig. S4K) caused by PPRV infection were abolished in the DFOM-treated cells. These results indicated that PPRV infection-induced ferroptosis was mediated by iron. Our results also revealed that DFOM significantly relieved the expression of reticulophagy-related proteins (Fig. S4L) and the colocalization of LC3-II puncta with the ER (Fig. S4M) induced by PPRV infection. To further analyze the role played by iron in PPRV infection-induced reticulophagy, EECs were transfected with small-interfering RNA (siRNA) targeting TFRC, an iron-carrying protein that is critical for iron transport into cells, followed by PPRV infection for the indicated times. The results revealed that inhibition of TFRC expression by siRNA attenuated reticulophagy-related protein expression (Fig. S4N) and the colocalization of LC3-II puncta with the ER (Fig. S4O) compared with mock-transfected EECs. Taken together, these results revealed that PPRV infection-induced reticulophagy and ferroptosis are mediated by cellular iron accumulation.

To analyze the role of ferroptosis in PPRV replication, PPRV infection assays were performed with EECs and GFFs treated with erastin or ferrostatin-1. After erastin treatment, the cell viability remains 50% remained at approximately 50% (30 μM) and 60% (20 μM), and therefore, 20 µM was used as the optimum concentration in subsequent experiments (Fig. S4P); after ferrostatin-1 treatment, the cell viability was not influenced at either 5 µM or 50 µM (Fig. S4Q). After treatment with erastin and ferrostatin-1, respectively, PPRV infection was examined by western blotting and TCID_50_ assays. Our data revealed that compared with DMSO, erastin increased the level of the PPRV N protein (Fig. 4O) and the virus titers (Fig. 4P) in a time-dependent manner. In contrast, the levels of PPRV N protein (Fig. 4Q) and virus titers (Fig. 4R) were decreased in ferrostatin-1-treated cells in a dose-dependent manner. In addition, to investigate whether reticulophagy is associated with enhanced PPRV replication induced by iron overload, the cells were transfected with siRNA targeting ATG5, cocultured with FAC, and then infected with PPRV. Our data revealed that the knockdown of ATG5 (Fig. 4S) expression significantly inhibited viral replication compared with that in the indicated control cells (Fig. 4S and T). Taken together, our results clearly revealed that iron overload facilitates viral replication by inducing excess lipid peroxidation-triggered ferroptosis and reticulophagy.

### PPRV infection enhances the localization of cellular iron on the ER and causes ER membrane damage by promoting excess lipid peroxidation to induce reticulophagy

**Fig 5:**
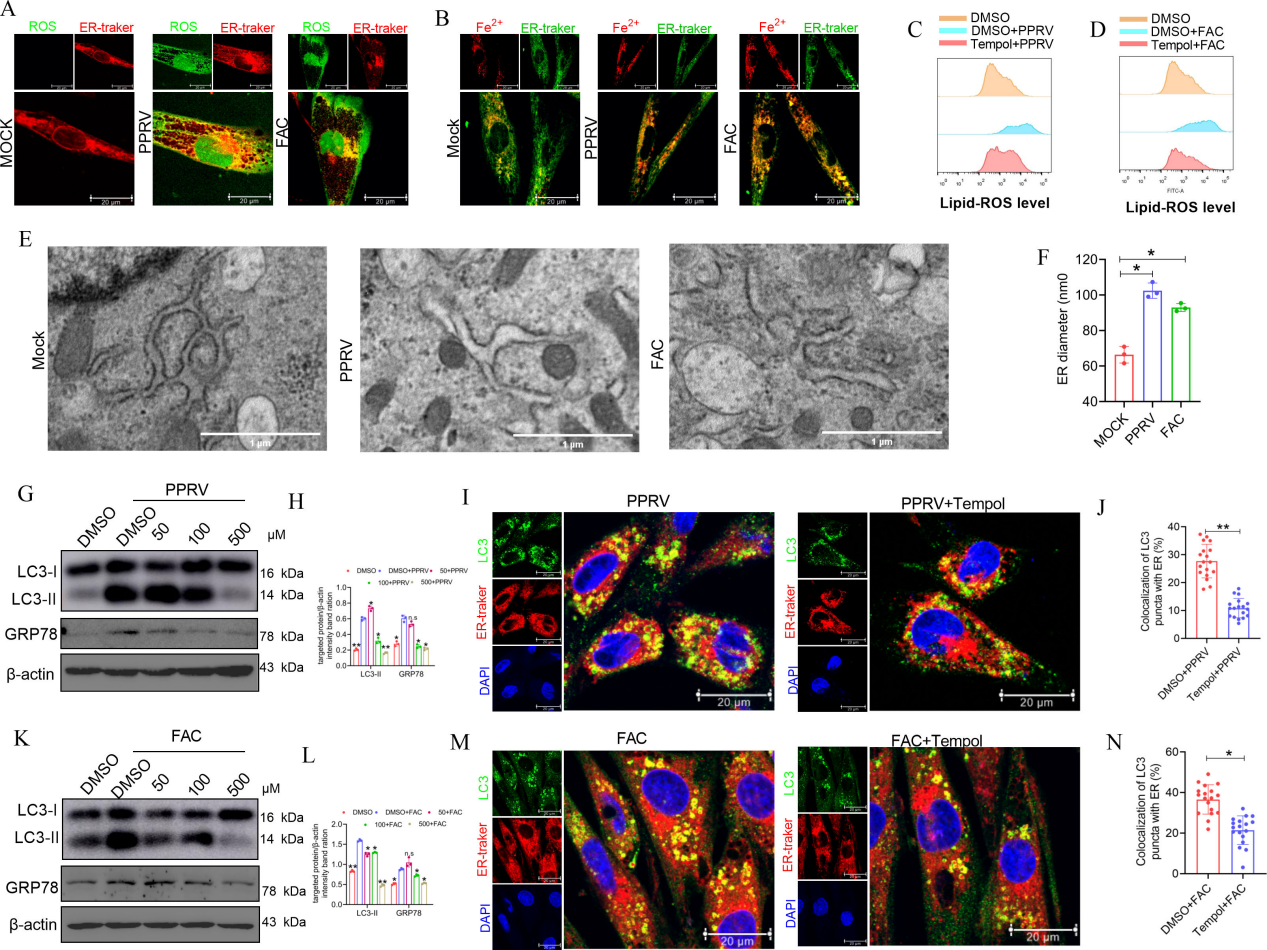
PPRV infection enhances the location of cellular iron on ER and causes ER membrane damage by promoting excess lipids peroxidation to induce reticulophagy. (A, B, E, and F) EECs were infected with PPRV (MOI = 3) or treated with FAC (100 µm) for 48 h. Then, (A) the colocalization between H_2_DCFDA-stained intracellular ROS (green) and ER tracker-labeled endoplasmic reticulum (red) were shown by confocal immunofluorescence microscopy, respectively. (B) The colocalization between FerroOrange-stained intracellular iron (red) and ER tracker labeled endoplasmic reticulum (green) and (E) the ultrastructural analysis of ER was conducted through TEM. Scale bar: 1 µm. (F) Endoplasmic reticulum width measurement. (C and D) EECs were treated with Tempol (100 µM) or DMSO for 2 h and then (C) infected with PPRV (MOI = 3) or (D) treated with FAC (100 µM) for 48 h. Then, the level of lipid ROS was detected by flow cytometry. (G and K) EECs were treated with different concentrations of Tempol or DMSO for 2 h and then (G) infected with PPRV (MOI = 3) or (K) was treated with FAC (100 µM) for 48 h. Then, the cells were subjected to western blot for the analysis of the expression of LC3 and GRP78. (H and L) The relative quantification of LC3 and GRP78 protein levels compared with β-actin protein levels was determined by densitometry. EECs were (I) treated with Tempol (100 µM) or DMSO for 2 h and then infected with PPRV (MOI = 3) or (M) treated with FAC (100 µM) for 48 h, and then, reticulophagy was determined by assessing the colocalization between LC3-positive autophagosomes (green) and ER tracker labeled endoplasmic reticulum (red). (J and N) The percentage of colocalization of LC3 puncta and ER. The cell nuclei were counterstained with DAPI. β-Actin was used as a loading control in western blot analysis. An asterisk indicates a comparison with the indicated control. *P < 0.05; **P < 0.01.

Iron promotes lipid peroxidation and produces excess lipid ROS, which causes membrane damage (30). We hypothesized that as a cellular organelle enriched with PUFA-PLs, the ER is the organelle with the highest rate of lipid peroxidation, resulting in lethal levels of lipid ROS-induced ERS and subsequent reticulophagy. Therefore, we first examined the location of ROS and the ER during PPRV infection or FAC-stimulated iron overload by confocal immunofluorescence microscopy. Compared with those in control cells, ROS were clearly produced, and some of these ROS colocalized with the ER in PPRV-infected or FAC-treated EECs (Fig. 5A), suggesting that lipid peroxidation occurred at the ER membrane during PPRV infection. Considering the important role of iron in lipid peroxidation, iron may translocate to the ER to catalyze lipid peroxidation. Therefore, cellular iron labeled with FerroOrange was subjected to immunofluorescence staining to assess its localization. As expected, our data demonstrated that larger amounts of cellular iron were localized on the ER in PPRV-infected and in FAC-treated EECs than in mock-treated cells (Fig. 5B). We also used DFOM to chelate iron and found that the translocation of iron to the ER induced by PPRV infection decreased after DFOM treatment (Fig. S5A). Additionally, TEM analysis revealed that PPRV infection induced significant expansion of the ER lumen (Fig. 5E and F), which was similar to the effect of FAC treatment on cells (Fig. 5E and F). Taken together, our results revealed that PPRV infection increased the localization of cellular iron at the ER, which catalyzes lipid peroxidation to cause ER membrane damage by promoting excess ROS production.

To verify the role of lipid ROS in PPRV infection or iron overload-regulated reticulophagy, tempol, a ROS scavenger, was used to eliminate excess lipid ROS produced after PPRV infection or FAC treatment. CCK-8 assays revealed that 100 µM tempol caused excess cell death (Fig. S5B). Therefore, 100 µM was used as the optimum concentration for tempol in subsequent experiments. The results revealed that tempol significantly decreased the amount of lipid ROS produced after PPRV infection (Fig. 5C) or FAC treatment (Fig. 5D) in EECs. Importantly, western blot analysis revealed that tempol treatment abolished the effects on the protein expression of GRP78 and LC3-II; moreover, immunofluorescence staining indicated that PPRV infection (Fig. 5I and J) or FAC (Fig. 5M and N) decreased the colocalization of LC3-II puncta with the ER. Together, these results suggested that lipid ROS production induced by PPRV infection-induced iron overload plays a key role in triggering reticulophagy.

### The lncRNA APR/miR-3955-5p/FTH1 axis inhibits PPRV infection-induced reticulophagy and ferroptosis by decreasing intracellular Fe^2+^ accumulation

**Fig 6:**
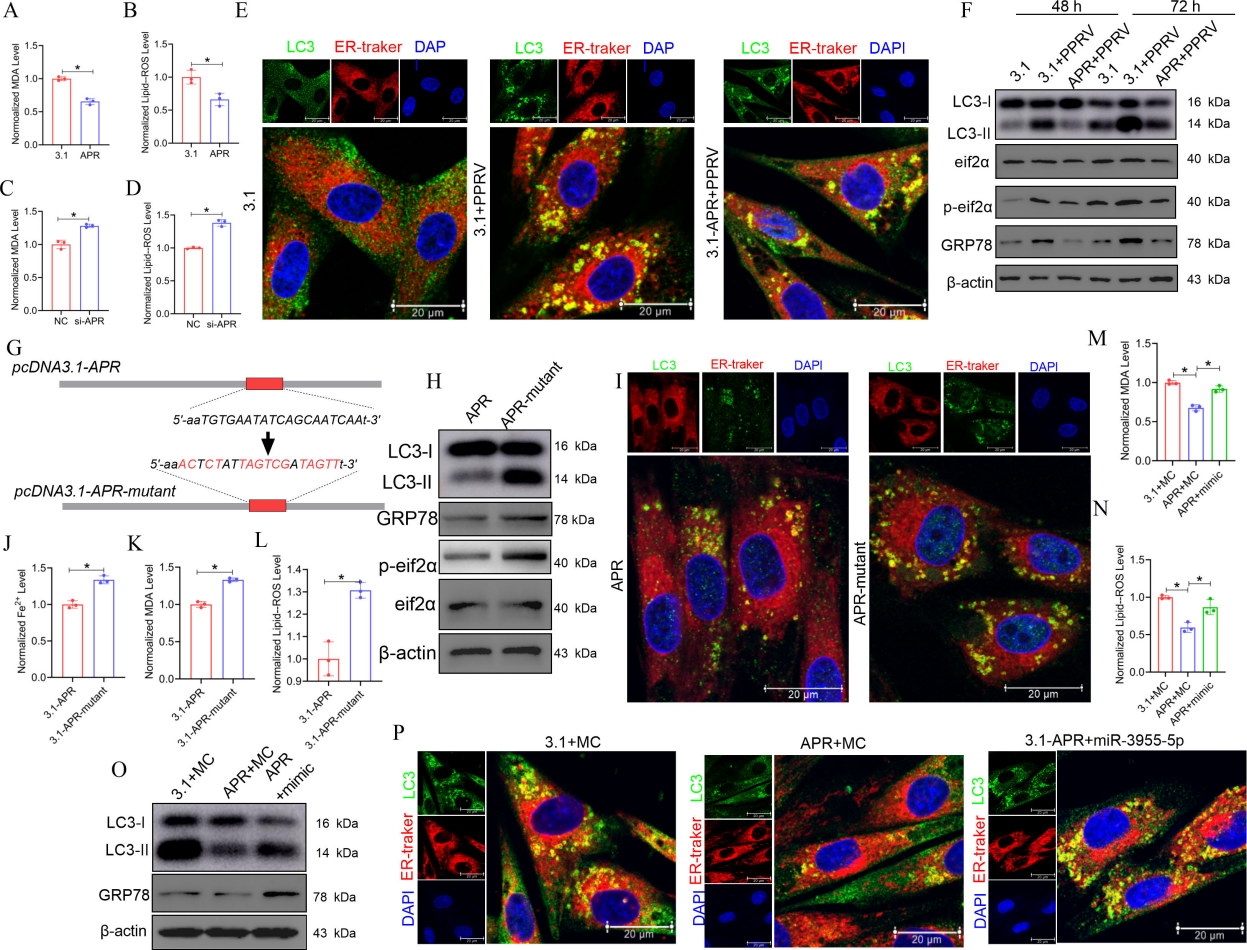
lncRNA APR/miR-3955-5p/FTH1 axis inhibits PPRV infection-induced reticulophagy and ferroptosis by decreasing intracellular Fe^2+^ accumulation. EECs were transfected with pcDNA3.1-APR (A and B) or si-APR (C and D) for 24 h and then infected with PPRV at an MOI of 3 for 48 h. Then, the levels of MDA (A and C) and lipid ROS (B and D) were detected. EECs were transfected with pcDNA3.1 or pcDNA3.1-APR for 24 h and then infected with PPRV at an MOI of 3. Forty-eight hours later, (E) reticulophagy was determined by assessing the colocalization between LC3-positive autophagosomes (green) and ER tracker labeled endoplasmic reticulum (red); meanwhile, (F) the cells were subjected to western blot for the analysis of the expression of the expression of LC3, phosphorylation of eIF2α, eIF2α, and GRP78. (G) Model of plasmid construction. (H–L) EECs were transfected with pcDNA3.1-APR-mutant and pcDNA3.1-APR respectively cells before PPRV infection. (H) Western blot for the analysis of the expression level of LC3, phosphorylation of eIF2α, eIF2α, and GRP78. (I) Reticulophagy was determined by assessing the colocalization between LC3-positive autophagosomes (green) and ER tracker labeled endoplasmic reticulum (red). (J) Level of MDA, (K) level of MDA, and (L) level of lipid ROS were detected. (M–P) EECs were cotransfected with pcDNA3.1 empty vector or pcDNA3.1-APR and control mimic (MC) or miR-3955-5p mimic for 24 h, and then, the cells were treated with FAC (100 µM). Forty-eight hours later, (M) the level of MDA and (N) lipid ROS were detected; meanwhile, (O) the cells were subjected to western blot for the analysis of the expression of the expression of LC3 and GRP78, and (P) reticulophagy was determined by assessing the colocalization between LC3-positive autophagosomes (green) and ER tracker labeled endoplasmic reticulum (red). The cell nuclei were counterstained with DAPI. β-Actin was used as a loading control in western blot analysis. An asterisk indicates a comparison with the indicated control. *P < 0.05; **P < 0.01.

Given the important role of iron overload in PPRV infection-induced reticulophagy and ferroptosis and the role of the lncRNA APR/miR-3955-5p/FTH1 axis in regulating cellular iron homeostasis, we explored whether APR plays a role in PPRV infection-induced ferroptosis and reticulophagy mediated via the miR-3955-5p/FTH1 axis. APR overexpression decreased the intracellular concentrations of Fe^2+^ (Fig. 3I), MDA (Fig. 6A), and lipid ROS (Fig. 6B) in PPRV-infected EECs. In contrast to APR overexpression, APR knockdown significantly increased the intracellular concentrations of Fe^2+^ (Fig. 3J), MDA (Fig. 6C), and lipid ROS (Fig. 6D) in PPRV-infected EECs. We also evaluated the effects of APR on PPRV infection-induced reticulophagy. Our immunofluorescence staining results revealed that the colocalization of LC3-II puncta with the ER was weak after APR overexpression in PPRV-infected EECs (Fig. 6E). Consistent with the immunofluorescence staining results, western blotting revealed that the protein expression of GRP78 and eIf2α and the phosphorylation levels of eIf2α and LC3-II were decreased after APR overexpression in PPRV-infected EECs after 48 h and 72 h (Fig. 6F). Taken together, these results indicated that APR decreases the sensitivity of EECs to PPRV infection-induced reticulophagy and ferroptosis.

To further explore whether APR plays a role in PPRV infection-induced ferroptosis and reticulophagy mediated via the miR-3955-5p/FTH1 axis, we analyzed the effects of miR-3955-5p on ferroptosis and reticulophagy in EECs infected with PPRV. The results suggested that the miR-3955-5p mimic increased the intracellular concentrations of Fe^2+^ (Fig. 3K), MDA (Fig. S6A), and lipid ROS (Fig. S6B). The miR-3955-5p inhibitor downregulated the protein expression of GRP78 and LC3-II (Fig. S6C) and the colocalization of LC3-II puncta with the ER (Fig. S6D). We subsequently designed several functional complementarity experiments to further investigate whether APR-regulated ferroptosis and reticulophagy are mediated by miR-3955-5p during PPRV infection. Our results suggested that the miR-3955-5p mimic reversed the inhibition of ferroptosis (Fig. 3L; Fig. S6E and F) and reticulophagy (Fig. S6G and H) resulting from APR expression in PPRV-infected cells. On the basis of the verified relationship between miR-3955-5p and FTH1, we assessed the role of FTH1 in PPRV infection-induced ferroptosis and reticulophagy. The results revealed that EECs overexpressing FTH1 after transfection with a pcDNA31-FTH1 plasmid before infection with PPRV exhibited tolerance to ferroptosis (Fig. 3M; Fig. S6I and J) and reticulophagy (Fig. S6K and L).

Moreover, a plasmid overexpressing the APR mutant lacking the ability to interact with miR-3955-5p (pcDNA3.1-APR-mutant) was constructed to further demonstrate the importance of the APR/miR-3955-5p/FTH1 axis in PPRV infection-induced ferroptosis, reticulophagy, and viral replication. Then, we transfected pcDNA3.1-APR-mutant and pcDNA3.1-APR, respectively, into cells before PPRV infection (Fig. 6G). As a result, we observed that transfecting cells with the pcDNA3.1-APR-mutant increased intracellular concentrations of Fe^2+^ (Fig. 6J), MDA (Fig. 6K), and lipid ROS (Fig. 6L). Moreover, cells transfected with the pcDNA3.1-APR-mutant presented greater expression of GRP78 and LC3-II (Fig. 6H) and more obvious colocalization of LC3-II puncta with the ER than did the pcDNA3.1-APR-transfected cells (Fig. 6I). Taken together, these results revealed that APR attenuates PPRV infection-triggered ferroptosis and reticulophagy by sponging miR-3955-5p to upregulate the protein expression of FTH1.

Next, we explored whether lncRNA APR/miR-3955-5p/FTH1 axis inhibits PPRV infection-induced reticulophagy and ferroptosis by decreasing intracellular Fe^2+^ accumulation. To test our hypothesis, we treated EECs with FAC after cotransfecting them with a pcDNA3.1 empty vector or with pcDNA3.1-APR and miRNA-3955-5p MC to establish the experimental group or after transfecting them with pcDNA3.1-APR and a miRNA-3955-5p mimic to establish the rescue group. Ferroptosis and reticulophagy in the cells were subsequently measured. The results indicated that the miRNA-3955-5p mimic abolished the APR-mediated inhibition of FAC-induced ferroptosis (Fig. 3O, 6M and N) and reticulophagy (Fig. 6O and P) in EECs. Importantly, EECs overexpressing FTH1 in the FAC treatment environment exhibited tolerance to ferroptosis (Fig. 3P; Fig. S6M and N). In addition, in FTH1 overexpressing EECs before FAC treatment, reticulophagy was decreased (Fig. S6O and P). Thus, we concluded that APR reduces the cellular iron level to attenuate iron overload-induced ferroptosis and reticulophagy through the miR-3955-5p/FTH1 axis.

### IRF1 positively regulates the activity of the APR promoter and promotes its transcription after PPRV infection

**Fig 7:**
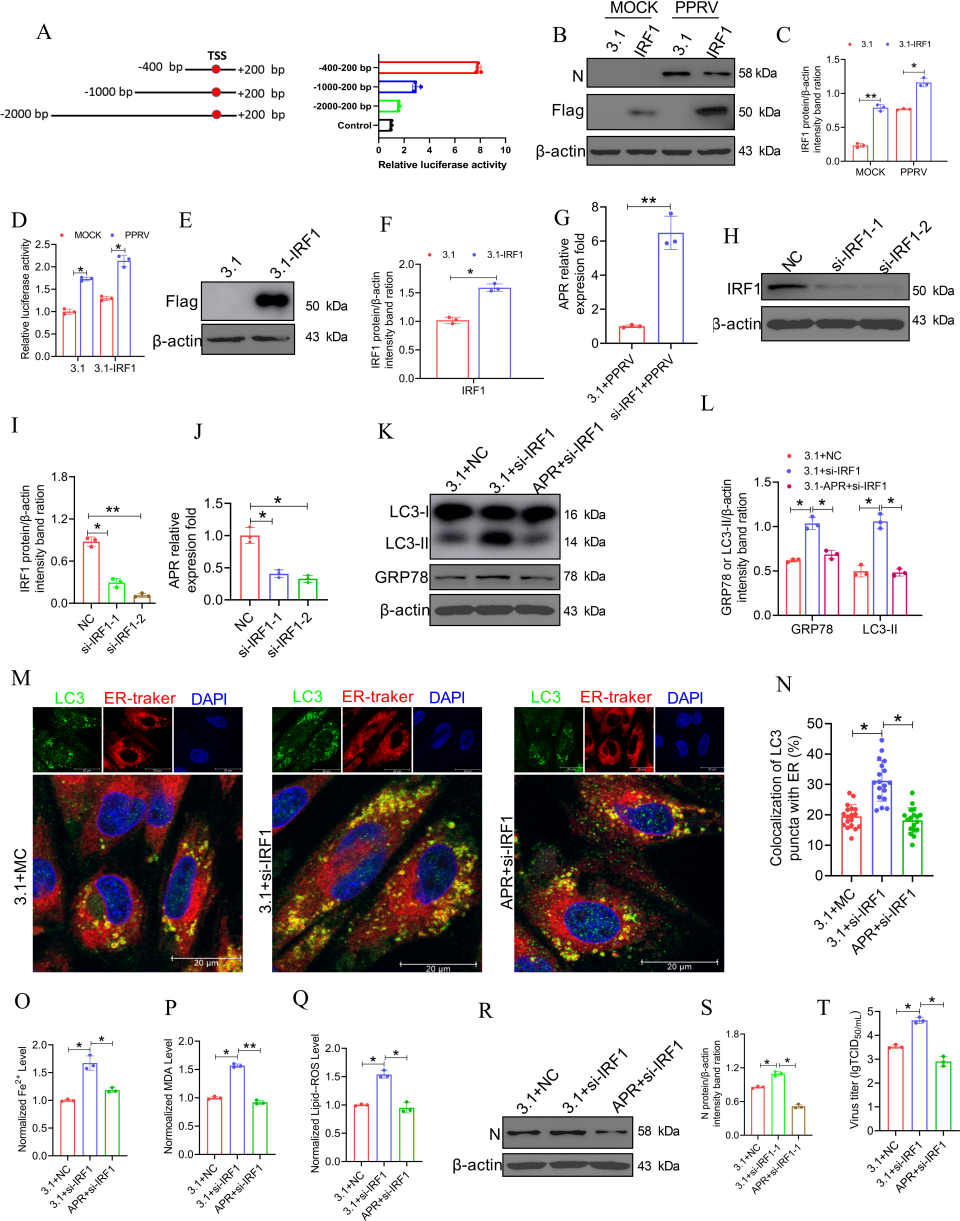
IRF1 positively regulates the activity of the APR promoter and promotes its transcription under PPRV infection. (A) EECs were co-transfected with a different region of APR promoter-reporter plasmids and phRL-TK, and then, the cells were harvested for dual-luciferase assays 48 h later. (B–D) The IRF1 overexpression EECs were co-transfected with pGL4.10 containing the region (−400/+200 bp) of APR promoter and phRL-TK before being infected with PPRV (MOI = 3). After 48 h infection, the cells were subjected to (B) western blot for the analysis of the expression of N and IRF1, and to (D) dual-luciferase assays for the test of luciferase activity. (C) The relative quantification of IRF1 protein levels compared with β-actin protein levels was determined by densitometry. (E and G) EECs were transfected with pcDNA3.1 or pcDNA 3.1-IRF1 for 24 h and then infected with PPRV at an MOI of 3 for 48 h. Then, the cells were subjected to (E) western blot for the analysis of the expression of IRF1 and (G) qRT-PCR for the analysis of the expression of APR. (F) The relative quantification of IRF1 protein levels compared with β-actin protein levels was determined by densitometry. (H and J) EECs were transfected with si-Control and siRNA against IRF1 (si-IRF1-1/2) for 24 h and then infected with PPRV at an MOI of 3 for 48 h. Then, the cells were subjected to (H) western blot for the analysis of the expression of IRF1, and (J) qRT-PCR for the analysis of the expression of APR. (I) The relative quantification of IRF1 protein levels compared with β-actin protein levels was determined by densitometry. (K–T) EECs were cotransfected with pcDNA3.1 empty vector or pcDNA3.1-APR and nonspecific control siRNA (si-Control) or siRNA against IRF1 (si-IRF1) for 24 h then infected with PPRV at an MOI of 3 for 48 h. Then, (K) the expression of LC3 and GRP78 was tested by western blot, (M) the reticulophagy was determined by assessing the colocalization between LC3-positive autophagosomes (green) and ER tracker labeled endoplasmic reticulum (red), the level of (O) ferrous iron, (P) MDA, and (Q) lipid ROS were detected, western blot (R) and TCID_50_ (T) assays were performed to determine the viral replication and progeny. (L and S) The relative quantification of indicated protein levels compared with β-actin protein levels was determined by densitometry. (N) The percentage of colocalization of LC3 puncta and ER. The cell nuclei were counterstained with DAPI. β-Actin was used as a loading control in western blot analysis. The data represent the mean ± SD of three independent experiments. *P* values were calculated using Student’s *t* test. An asterisk indicates a comparison with the indicated control. *P < 0.05; **P < 0.01.

To investigate the regulatory transcription mechanism of APR, we analyzed the promoter activation of APR during PPRV infection. A series of progressively truncated luciferase reporter constructs (pGL4.10-APR-) (from bp positions −2000 to +200) were transiently transfected into EECs. The region between −400 and +200 bp presented the highest transcriptional activity, suggesting that this region contains the core promoter sequence (Fig. 7A); that is, the −400 and +200 bp region was identified as the region harboring key active sites. The sequence was subsequently subjected to analysis with the bioinformatic tools in the JASPAR and Gene Regulation databases to identify transcription factors that bind between the −400 and +200 bp region. An IRF1-binding site was identified in this region. To determine whether IRF1 regulates the transcription of APR, we cotransfected pGL4.10-APR (carrying the sequence from the −400 to +200 bp region) and 3.1-IRF1 or an empty plasmid into EECs and then infected the cells with PPRV. After 48 h in culture, the EECs were subjected to a dual-luciferase reporter assay. The results revealed that the overexpression of IRF1 increased the luciferase activity of reporter constructs carrying the predicted IRF1-binding sites (the 400 to +200 region bp sequence) not only in infected cells but also in mock-infected cells (Fig. 7B through D). Moreover, IRF1 overexpression increased (Fig. 7E through G), whereas IRF1 knockdown (Fig. 7H through J) significantly decreased the transcription of endogenous APR. In addition, we found that IRF1 mediated APR-regulated reticulophagy (Fig. 7K through N), ferroptosis (Fig. 7O through Q) and viral replication (Fig. 7R through T). Collectively, these results suggested that IRF1 positively regulates the activity of the APR promoter and thus promotes its transcription.

## DISCUSSION

Iron is an essential element and a critical component of molecules involved in many biological processes. Despite an increasing number of studies on the involvement of iron in viral pathogenesis and even cell fate (27–29, 53), the role of iron in infection by the viruses of *Morbillivirus* genus and in the induction of autophagy during PPRV infection has largely been unexplored. In the present study, we revealed, for the first time, that PPRV infection increased the level of iron and that increase in cellular iron further triggered ferroptosis and reticulophagy by promoting the peroxidation of ER membrane-bound lipids. Importantly, our results indicated that cellular iron and iron-mediated ferroptosis and reticulophagy enhance PPRV replication. In addition, we identified a host antiviral lncRNA, APR, that attenuates PPRV infection-induced reticulophagy and ferroptosis by sponging miR-3955–5, a negative miRNA that directly targets the gene encoding the FTH1 protein, which stores iron and ultimately alters cellular iron homeostasis. Furthermore, we found that APR is regulated at the transcriptional level by IRF1; that is, IRF1 enhances APR transcription.

As a newly discovered form of regulated cell death, ferroptosis has been intensively studied in a wide range of human cancer models, which show variable sensitivity to its induction (54, 55). However, the effects of viruses on lipid ROS production and ferroptosis have rarely been studied. Nevertheless, studies reveal relationships between viruses, such as NDV, HCV, EBV, IAV, CV, and HBV (40, 42, 43, 56–58); ferroptosis has been reported, and the number of studies has gradually increased in recent years. Our data indicated that PPRV significantly sensitizes host cells to iron-dependent ferroptosis. Unfortunately, we only studied the role of the virus as a whole in ferroptosis. However, viruses can induce cell death through their components, such as nucleic acids or viral proteins (46, 59, 60), which prompts us to focus on the role of PPRV viral proteins or RNA in ferroptosis in future studies. Although cell death is thought to be a strategy for host defense against virus infection (61), mounting evidence indicates that cell death is beneficial for the release and spread of viruses. Our previously published work demonstrated that autophagy contributes to PPRV infectivity (46, 62). In the present study, our results revealed that another type of cell death, ferroptosis, also promotes viral replication. Collectively, the advances we have made in study PPRV in recent years might demonstrate that PCD is closely related to the PPRV life cycle.

Autophagy is an evolutionarily conserved degradation process and can be classified into either a nonselective or a selective process. Selective autophagy is an intracellular quality control mechanism that selectively mediates the degradation of specific targets, such as invading pathogens, aggregated proteins, and damaged organelles (22). Our previously published study revealed that PPRV infection induces two successful and distinct waves of autophagy and that ERS induced by PPRV can mediate the induction of autophagy to promote viral replication (46, 47). In the present study, PPRV infection triggered reticulophagy, which removes unwanted or damaged portions of the ER. In selective autophagy, autophagy receptors bind to cargoes and trigger cargo degradation within lysosomes/vacuoles. To date, seven autophagic cargo receptors, cell cycle progression 1 (CCPG1), reticulophagy regulator 1 (RETREG1/FAM134B), reticulon 3 (RTN3), SEC62 homolog, preprotein translocation factor (SEC62), testis expressed 264, ER-phagy receptor (TEX264), atlastin GTPase 3 (ATL3), SEC24 homolog C, and COPII coat complex component (SEC24C) have been found to specifically bind to the ER and autophagosomal membranes, thereby contributing to reticulophagy selectivity (63). In this study, we demonstrated that FAM134B, located in the ER, is degraded by the autophagic pathway during PPRV infection, suggesting that this molecule is responsible for PPRV-induced reticulophagy. In our previously published study, PPRV N and C were demonstrated to be two important viral components that contribute to the occurrence of autophagy. Therefore, we speculate that N and C might interact with FAM134B to promote reticulophagy, but this need to be further studied.

Disruption of iron homeostasis is linked to infection, cancer, and aging-related diseases (25, 26, 64). Viral infection changes the cellular iron level; in turn, cellular iron influences viral replication, exerting different effects. For example, a high concentration of iron inhibits the replication of hepatitis C virus (HCV), herpes simplex virus 1 (HSV-1), and BVDV (27). In contrast, iron removal by chelators inhibits the replication of HIV-1, West Nile virus (WNV), and human cytomegalovirus (HCMV) (28, 65, 66). In this study, our results suggested, for the first time, that cellular iron homeostasis is disrupted during PPRV infection. Furthermore, we demonstrated that the disruption of the balance of cellular iron metabolism is mediated by the upregulation of the TFRC protein by PPRV infection. However, to determine how PPRV induces TFRC-mediated iron overload, further exploration is needed. Additionally, we found that iron overload increased PPRV replication via ferroptosis and reticulophagy. Notably, iron homeostasis influences the immune system, including innate and adaptive immunity (64), and our previous work demonstrated that PPRV induces immune suppression, which promotes its replication (7). In this study, we found that iron overload-mediated lipid peroxidation and its products regulate type I IFN response to influence its propagation. In other words, during the process of ferroptosis and reticulophagy, lipid peroxides produced by lipid peroxidation suppress antiviral response and then enhance PPRV replication. Additionally, that is the reason why ferroptosis and reticulophagy could regulate virus replication. However, the specific mechanism of iron overload affects antiviral response through lipid peroxidation needs to be further investigated.

Iron can participate in the peroxidation of membrane-bound lipids, which subsequently results in membrane damage (34). In addition, as an intracellular organelle enriched with membrane-bound lipids, the ER is an essential site of lipid peroxidation (32). Given that our previous work demonstrated that PPRV infection induces ERS (47) and that lipid peroxidation of membrane-bound lipids in the ER, enhanced by iron overload, is believed to lead to a breakdown in the membranes of the ER (35, 36) and can even elicit ERS (37, 38), we focused on PPRV infection-induced iron overload in PPRV infection-induced ERS and subsequent reticulophagy. Our data demonstrated, for the first time, that PPRV infection promotes ERS and then reticulophagy by increasing the iron level, translocating iron to the ER, and catalyzing the peroxidation of ER membrane-bound lipids, ultimately damaging the ER via lethal levels of ROS production. Additionally, the enhanced localization of iron on ER indicates where the iron transported from the extracellular flows toward.

Recently, increasing evidence has demonstrated that ferroptosis is an autophagy-dependent form of cell death (67–73). In particular, the role of selective autophagy, such as ferritinophagy, lipophagy, clockophagy, and chaperone-mediated autophagy (CMA), in promoting ferroptosis has been explored by many researchers (22). For example, NCOA4-dependent ferritinophagy degrades the iron-storing protein ferritin, which is critical for the regulation of the cellular iron level and promotes ferroptosis (68). Although studies have provided novel insight into the interplay between autophagy and ferroptosis, they seem to focus more on the role of iron in ferroptosis. Therefore, we investigated whether intracellular iron overload can regulate autophagy and found, for the first time, that the increase in iron levels induced by PPRV infection induces not only ferroptosis but also reticulophagy, a type of selective autophagy. Notably, this is the first study to demonstrate that iron can induce reticulophagy. Although ERS is related to ferroptosis in some cases (74), we still cannot determine whether the reticulophagy induced by PPRV infection plays a role in ferroptosis. Therefore, the functional role of reticulophagy in ferroptosis needs to be further explored.

lncRNAs contribute to gene regulation through diverse mechanisms, including guiding or sequestering chromatin-modifying enzymes and transcriptional complexes in the nucleus; regulating mRNA processing, splicing, and translation; and acting as competitive inhibitors of endogenous RNAs (e.g., microRNAs) or proteins in the cytoplasm (10). Therefore, the subcellular localization of lncRNAs is a leading determinant of their molecular function (75). Hence, given that APR is located in both the cytoplasm and nucleus under normal physiological conditions but is located mainly in the cytoplasm during PPRV infection, we investigated the underlying mechanism by which APR in the regulation of regulates reticulophagy and ferroptosis from the perspective of its ceRNA action. Although we revealed an underlying mechanism for APR located in the cytoplasm in the regulation of reticulophagy and ferroptosis, how APR functions in the nucleus needs to be further studied. In addition, lncRNAs regulate gene expression by interacting with not only RNAs but also proteins (RNA-binding proteins [RBPs]) (76), which have attracted increasing attention. Therefore, elucidating the mechanisms by which RBPs mediate APR functions during PPRV infection will be the focus of our next study.

After observing the abnormal transcription of APR and its significant role, we investigated the mechanisms through which APR is regulated at the transcriptional level by analyzing the promoter activation of APR after PPRV infection. Interestingly, we identified IRF1 as the transcription factor critical for the PPRV-mediated transcriptional upregulation of APR. IRF1 is an extensively characterized interferon-stimulated gene (ISG) and a central regulator of the IFN response. In fact, our previous work revealed a relationship between IRF1 and PPRV infection and demonstrated that PPRV infection upregulated IRF1 expression to inhibit viral replication by promoting ISG production (16). In this study, our data suggested that upregulated IRF1 can inhibit viral proliferation, even reticulophagy and ferroptosis, by increasing the transcription of APR during PPRV infection. Therefore, our present study and previous studies together reveal that IRF1 plays an antiviral role in PPRV infectivity in different ways.

**Fig 8:**
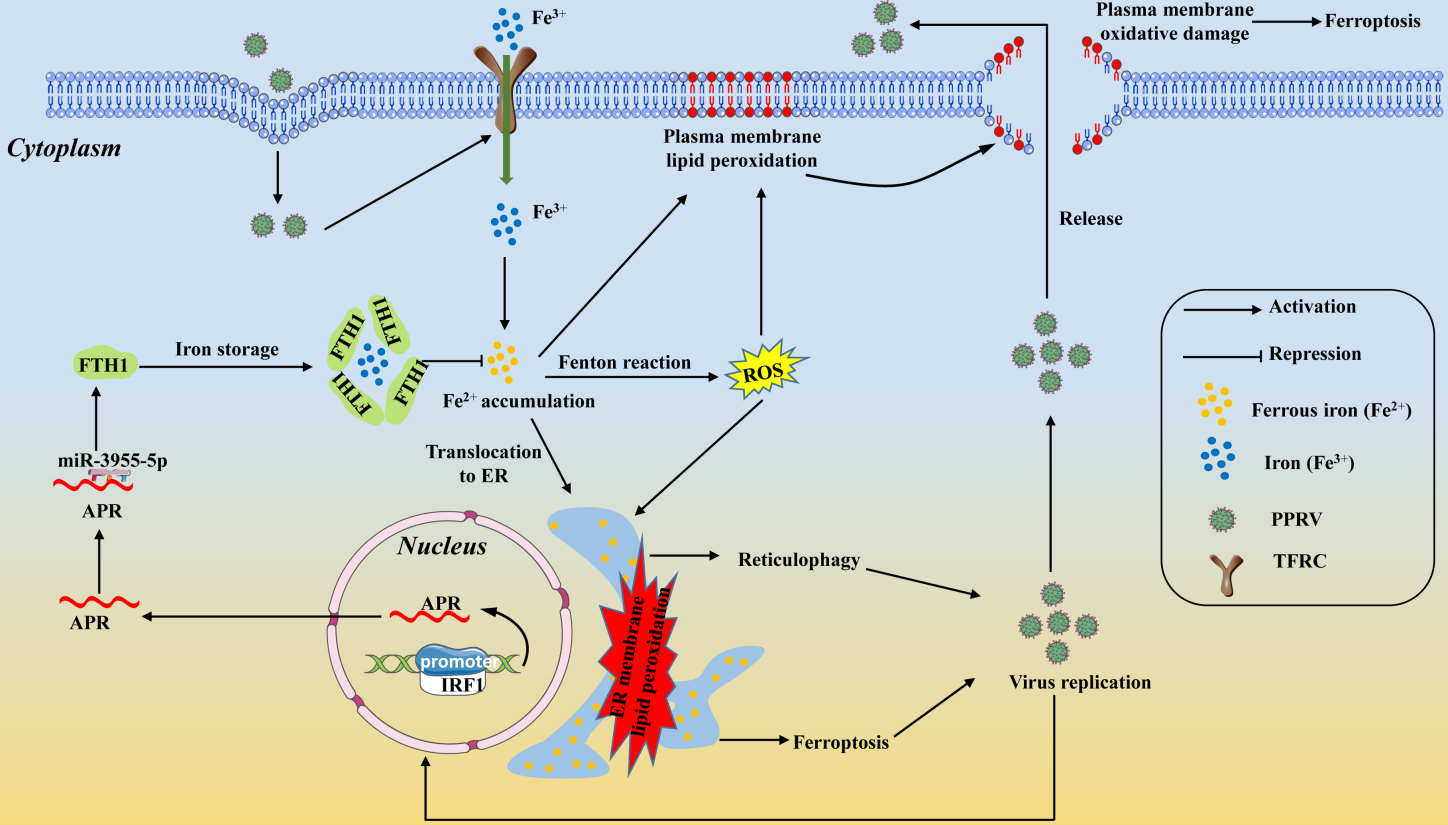
Proposed model for long noncoding RNA APR attenuates PPRV infection-induced intracellular iron accumulation to inhibit membrane lipid peroxidation and inhibits viral replication. PPRV infection increases cellular iron accumulation by upregulating TFRC expression, and more importantly, iron overload benefits viral replication as well as promotes ER membrane lipid peroxidation by enhancing the location of cellular iron on the ER and finally induces ferroptosis and reticulophagy. While, APR, a host antiviral lncRNA, is induced to decrease PPRV infection-induced intracellular Fe^2+^ accumulation via miR-3955-5p/FTH1 axis and finally inhibits reticulophagy and ferroptosis. Additionally, IRF1 promotes APR transcription by positively regulating the activity of the APR promoter during PPRV infection.

In summary, we provided a novel model for the interaction between PPRV and host cells, which involves noncoding RNA regulation, iron homeostasis, and iron-related membrane lipid peroxidation. The model is shown in Fig. 8. On one hand, PPRV infection increases cellular iron accumulation by increasing TFRC expression, and more importantly, iron overload benefits viral replication promotes ER membrane lipid peroxidation by enhancing the localization of cellular iron on the ER and ultimately induces ferroptosis and reticulophagy. On the other hand, APR, a host antiviral lncRNA, is induced to decrease PPRV infection-induced accumulation of intracellular Fe^2+^ a via the miR-3955-5p/FTH1 axis and ultimately inhibits reticulophagy and ferroptosis. Additionally, IRF1 promotes APR transcription by positively regulating the activity of the APR promoter during PPRV infection. Overall, this study provides new insights into our understanding of the PPRV-host cell interactions and pathogenesis and offers potential therapeutic targets for antiviral intervention.

## MATERIALS AND METHODS

### Cell lines and virus

Caprine EECs were immortalized by transfection with human telomerase reverse transcriptase (hTERT), and we have previously confirmed that the secretory function of these cells is consistent with that of primary EECs (77, 78). EECs and primary GFF cells were cultured in Dulbecco’s modified Eagle medium/F-12 Ham’s medium (DMEM/F12; Gibco, Carlsbad, CA, USA) supplemented with 10% fetal bovine serum (Gibco, Carlsbad, CA, USA), 100 IU/mL penicillin, and 10 µg/mL streptomycin (Gibco, Carlsbad, CA, USA) at 37°C in 5% CO_2_. Virus stock was prepared by collecting the infected Vero cell supernatants when cytopathic effect (CPE) affected about 80% of the cells. The virus was harvested by three cycles of freezing and thawing and stored at −80°C and purified by banding on a sucrose gradient. The purified virus titers were estimated by estimating 50% tissue culture infective doses (TCID_50_) using Vero cells in 96-well microtiter plates. (TCID_50_ assays were also conducted for testing the virus titers in other experiments of this study based on the supernatants of the virus-infected cells.) The purified virus was tested for its infectivity in Vero cells and was used further for infection in EECs or GFFs. According to the requirements of the different experiments, EECs were infected with PPRV at an MOI of 3 or mock-infected with phosphate-buffered saline (PBS; 8.4 mM Na_2_HPO_4_, 1.5 mM KH_2_PO_4_, 136.9 mM NaCl, and 2.7 mM KCl) at 37°C for the indicated durations.

### Antibodies and reagents

Anti-PPRV-N monoclonal antibody was provided by the China Animal Health and Epidemiology Center (Qingdao, China). Anti-TFRC (sc-65882), anti-FSP1 (sc-377120), anti-NOX2 (sc-130543), and anti-ACSL4 (sc-365230) were all obtained from Santa Cruz Biotechnology. Anti-GPX4 (67763-1-Ig), anti-SLC7A11 (26864-1-AP), and anti-GRP78 (11587-1-AP) were purchased from Proteintech. Anti-eIF2α (ab169528), anti-phospho-eIF2α (ab32157), and anti-FTH1 (ab183781) were from Abcam. Anti-LC3-II B (L7543), HRP-conjugated goat anti-mouse IgG (A9917), and HRP-conjugated goat anti-rabbit IgG (A0545) were from Sigma. Anti-β-actin (HC201) and FITC-conjugated goat anti-rabbit IgG (HS211-01) were from TransGen. Iron Assay Kit (ab83366) was purchased from Abcam. C11-BODIPY (DSB61) was purchased from Thermo Fisher Scientific. GSH/GSSG ASSay kit (S0053), MDA ASSay kit (S0131S), ROS Assay Kit (S0033S), ER-Tracker Red (C1041), and ER-Tracker Green (C1042S) were purchased from Beyotime. FerroOrange (FerroOrange) was from dojindo.

### RNA isolation and real-time PCR analysis

TRizol reagent was used to extract the total RNA of goat cells according to the manufacturer’s instructions (Invitrogen, Waltham, MA, USA). Reverse transcription was carried out using M-MLV reverse transcriptase (Invitrogen, Waltham, MA, USA). qRT-PCR was performed using the SYBR Green master mix (TransGen Biotech, China) to quantify the RNA copy numbers on an iQ5 qRT-PCR System (Bio-Rad, US). The PCR cycling conditions were 2 min at 95°C followed by 40 cycles of 15 s at 94°C and 30 s at 60°C. Relative abundance of lncRNA and mRNA transcripts were analyzed and calculated by the threshold cycle method (2^−ΔΔCt^). The relative expression level of each gene was normalized to housekeeping gene β-actin. All specific primers used in this study are listed in Table S3.

Additionally, for the detection of miRNA, total RNA was reverse transcribed, and qRTPCR analysis was performed using stem-loop qRT-PCR. Stem-loop RT primers and probes for detecting miRNAs were designed based on their sequences (Table S4), and the sequences of the primer and TaqMan probe are not shown because of intellectual property protection. TaqMan MicroRNA RT kit (Applied Biosystems) was used, and the reaction mixtures were incubated according to kit instructions. Each 10 µL miRNA PCR included 1 µL of RT product, 5 µL of 2× IQ Supermix (Bio-Rad), 1.5 mM forward primer, 0.7 mM universal reverse primer, and 0.2 mM TaqMan probe (Applied Biosystems). The samples were incubated on an ABI 7500 system (Applied Biosystems, Warrington, UK) at 95°C for 3 min, followed by 40 cycles of 95°C for 15 s and 60°C for 30 s. All real-time PCRs were performed from the same batch of RT product for each sample and were performed in duplicate. TaqMan microRNA assay for U6 (Applied Biosystems) was used as a loading control.

### Transient transfections and chemical treatment

All plasmid and siRNA (Ribo, Gaungzhou, China) transfections were performed using TurboFect Transfection Reagent (Thermo Fisher Scientific, R0531) according to the manufacturer’s protocol. Various chemical reagents were used to pre-treat cells, including erastin (MCE, HY-15763), ferrostatin-1 (MCE, HY-100579), ammonium iron (III) citrate (MCE, HY-B1645), deferoxamine mesylate (MCE, HY-B0988), and Tempol (MCE, HY-100561). All the above treatments were applied 2 h before virus infection or other treatment and maintained until the end of the experiments.

### Western blot analysis

The harvested cells were treated with RIPA lysis buffer containing phenylmethyl sulfonyl fluoride (PMSF) for generating cell lysates. Protein samples were produced by adding 5× sodium dodecyl sulfate-polyacrylamide gel electrophoresis (SDS PAGE) sample buffer to lysed cells. The samples were boiled for 10 min, separated by 12% SDS-PAGE, and then transferred onto 0.22 µm polyvinylidene difluoride membranes (Millipore, Billerica, MA). The membranes were blocked with 5%–10% nonfat milk in Tris buffered saline with Tween-20 (TBST) for 2 h and then probed overnight at 4°C with primary antibodies. After washing, the membranes were reacted with HRP-conjugated secondary antibodies at room temperature for 1 h. At last, the bound antibodies were detected with enhanced chemiluminescence (ECL) immunoblotting detection reagents (Millipore, Billerica, MA). Images were obtained with a CanoScan LiDE 100 scanner (Canon).

### Transmission electron microscopy (TEM)

The cells were washed three times with phosphate buffer solution (PBS) and collected by centrifugation at 800 × *g* for 5 min. One drop of 2% preheated agarose was added to the cell pellet and uniformly mixed. After solidification, the agar was cut into 1 mm^3^ blocks and placed into a 2.5% glutaraldehyde/0.1 M phosphate buffer solution (Na_2_HPO_4_ · 12H_2_O, NaH_2_PO_4_ · 2 H_2_O, pH 7.4) at 4°C for fixation; the sections were then washed three times with PBSfor 10 min and post-fixed in 1% osmium tetroxide at 4°C for 1 h. Following dehydration with a graded series of ethanol solutions, the cells were embedded in a mixture of Epoxy 812 and warmed at 35°C, 45°C, and 60°C for 12 h, 12 h, and 48 h, respectively. Ultra-thin sections were prepared, stained with 4% uranyl acetate, and observed under a transmission electron microscope.

### Confocal immunofluorescence microscopy

After the relevant treatment, the cells were washed four times with PBS and fixed in 4% paraformaldehyde. The cells were washed again four times with PBS and treated with 0.1% Triton X-100 (Sangon Biotech, A110694) for 15 min. Then, the cells were incubated with 1% bovine serum albumin (BSA; Sigma-Aldrich, A7906), and the appropriate primary antibodies for 1 h at 37°C before being washed and incubated simultaneously with FITC- or PE-conjugated secondary antibodies. Finally, the cells were treated with a 2-(4-amidinophenyl)-6-indolecarbamidine dihydrochloride (DAPI) (Sigma-Aldrich, B2261) solution for 5 min and analyzed under a confocal microscope (CLSM; LeicaSP8, Germany)

### Cell viability assay

Briefly, EECs were seeded in 96-well cell culture plates with 200 µL DMEM/F12 containing 2% FBS at a density of 1 × 10 ^4^ cells per well. After incubation at 37°C in 5% CO _2_ for 24 h, different pharmacological inhibitors at the indicated concentrations were added and incubated for 24 h. Twenty microliters of MTT solution (Sigma-Aldrich Co.) was subsequently added and incubated for 4 h to allow the MTT to be fully metabolized. Then, the medium was removed, and the cells were washed with DMEM/F12. Finally, the cells were resuspended in formazanin 100 µL of DMSO solution, and the optical density was read at 540 nm.

### Quantification of ROS and lipid ROS

For the detection of ROS, 50 µM H_2_DCFDA was incubated with cells for 30 min at 37°C. For lipid ROS, the cells were incubated with 10 µM of C11-BODIPY for 30 min at 37°C, 5% CO_2_ and protected from light. Excess C11-BODIPY or H2DCFDA was removed by washing the cells with PBS twice. Subsequently, the cells were washed three times with PBS and analyzed by flow cytometry.

### Iron, GSH/GSSG, and MDA assays

#### Iron assays

For the iron assay, we used an Iron Assay Kit (Abcam, ab83366) to measure Fe^2+^ or Fe in each cell line. The cells were collected and washed in ice-cold PBS and homogenized in 5× volumes of iron assay buffer on ice, then centrifuged (13,000 × *g*, 10 min) at 4°C to remove insoluble material. To measure Fe^2+^, 5  µL of iron assay buffer was added to each well and two sets of wells were set up. Then, 5  µL of assay buffer was added to the samples in one set of wells, and 5  µL of iron reducer was added to the other set of wells. The level of ferric iron was calculated by subtracting ferrous iron from total iron. To measure total Fe, 5  µL of iron reducer was added to each sample well to reduce Fe^3+^ to Fe^2+^. Samples were mixed well using a horizontal shaker or by pipetting and the reactions were incubated for 30  min at room temperature in dark conditions. Then, 100  µL of iron probe was added to each well-containing standard or test samples. Samples were mixed well using a horizontal shaker or by pipetting, and the reactions were incubated for 60  min at 37°C in dark conditions. Finally, the absorbance was measured at 593  nm (*A*_593_).

#### GSH/GSSG assays

The intracellular GSH levels were measured by GSH and GSSG assay kit (Beyoyime, S0053). Cells were cultured in six-well plates and after different treatments, the samples were subjected to two rapid freeze-thaws using liquid nitrogen and a 37°C water bath. The supernatants were centrifuged and used for GSH measurements. According to the manufacturer’s instructions, the supernatant to be tested, GSH clear auxiliary solution, and total GSH assay working solution were added sequentially. The reaction was carried out at 25°C for 1 h. NADPH (0.5 mg mL^−1^) was added, the reaction was carried out at 25°C for 25 min, and the absorbance at 405 nm was measured using a microplate reader to calculate the GSH concentration.

#### MDA assays

The lipid peroxidation levels were measured using a colorimetric reaction based on the reaction of MDA and thiobarbituric acid (TBA) to produce a red product. Lipid Peroxidation MDA assay kit (Beyotime, S0131S) instructions were followed exactly; cell lysates from different treatments or adenovirus-infected lung tissue homogenates were mixed with MDA assay solution, heated at 100°C for 15 min, cooled to room temperature, and centrifuged at 1,000 × *g* for 10 min. The supernatant was then collected, and absorbance was measured at 450 nm using a microplate reader.

### Dual-luciferase reporter assays

To test the interaction of miR-3955-5p with APR or FTH1 mRNA, the plasmids of wild-type APR (APR-WT) and FTH1 (FTH1-WT) and mutant APR (APR-MUT) and FTH1 (FTH1-MUT) were designed and inserted into pmirGLO vectors (Promega, Madison, WI, USA). HEK293T cells were cultured onto 96-well plates at 10,000 cells per well overnight, then co-transfected with plasmids of dual-luciferase reporters and miRNA mimics or NC, detecting dual-luciferase activity by using the Dual-Glo Luciferase Assay System Kit (Promega, E2920) according to the manufacturer’s instructions. Firefly luciferase activity was normalized to renilla luciferase activity.

For APR promoter-luciferase reporter assays, the APR promoter sequence was analyzed using Ensembl and NCBI. Three progressive truncated fragments containing from −2000 to +200 relative to the transcription initiation site were cloned into the luciferase reporter vector pGL4.10 (Promega, E665A). EECs were co-transfected with luciferase constructs and the pRLTK vector (Promega, E2241). The cells were harvested 36–48 h after transfection and were detected using the dual luciferase reporter assay system.

### Statistical analysis

All values are expressed as the arithmetic mean of triplicates ± standard deviation (SD) from three independent experiments. Significance was determined by one-way analysis of variance (ANOVA) with a Dunnett post-test or by the Student’s *t* test. Values of *P* < 0.05 were considered to indicate statistical significance.

## SUPPLEMENTAL MATERIAL

Supplemental figures mbio.00127-25-s0001.docx Figures S1 to S6.

Table S1 mbio.00127-25-s0002.docx Differential expression of lncRNAs in PPRV‑infected when compared mock‑infected EECs cells.

Table S2 mbio.00127-25-s0003.docx lncRNA APR sequence.

Table S3 mbio.00127-25-s0004.docx qRT-PCR primers used in this study.

Table S4 mbio.00127-25-s0005.docx Sequences of the examined miRNAs analyzed in this study.

ASM does not own the copyrights to Supplemental Material that may be linked to, or accessed through, an article. The authors have granted ASM a non-exclusive, world-wide license to publish the Supplemental Material files. Please contact the corresponding author directly for reuse.
