# Supplementary material for: The long noncoding RNA APR attenuates PPRV infection-induced accumulation of intracellular iron to inhibit membrane lipid peroxidation and viral replication
Source: mBio. 2025 Mar 24;16(4):e00127-25. doi: 10.1128/mbio.00127-25 (PMC11980570; doi:10.1128/mbio.00127-25)
Supplement: Supplemental figures — Figures S1 to S6. [file mbio.00127-25-s0001.docx]

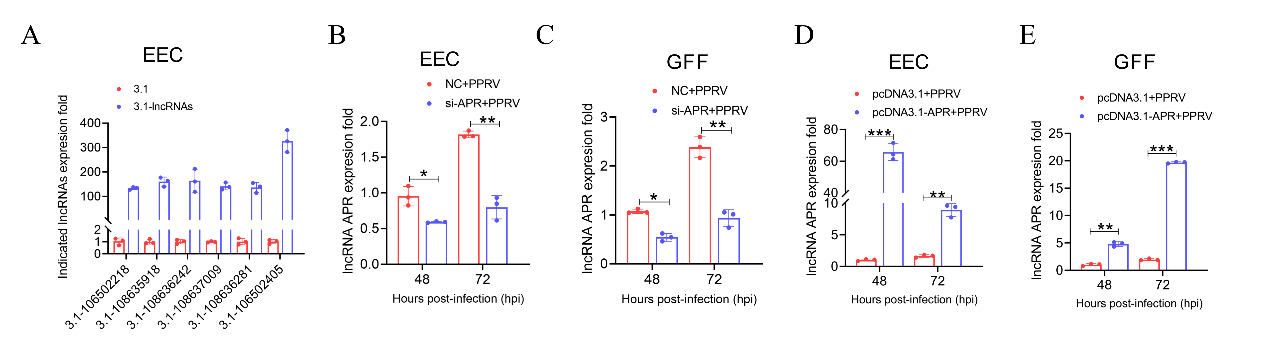


**Fig. S1.** (**A**) EECs were transfected with pcDNA3.1 or pcDNA 3.1-lncRNAs for 24 h and then infected with PPRV at an MOI of 3 for 48 h and 72h. Then, the cells were subjected to qRT-PCR for the analysis of the expression of six lncRNAs in EECs (**B** and **C**) EECs and GFFs were transfected with nonspecific control siRNA (NC) and siRNA against APR (si-APR) for 24 h and then infected with PPRV at an MOI of 3 for 48 h and 72h. Then, the cells were subjected to qRT-PCR for the analysis of the expression of APR in (**B**) EECs and (**C**) GFFs. (**D** and **E**) EECs and GFFs were transfected with pcDNA3.1 or pcDNA 3.1-APR for 24 h and then infected with PPRV at an MOI of 3 for 48 h and 72h. Then, the cells were subjected to qRT-PCR for the analysis of the expression of APR in (**D**) EECs and (**E**) GFFs. The data represent the mean ± SD of three independent experiments. *P* values were calculated using Student's *t* test. An asterisk indicates a comparison with the indicated control. **P* < 0.05; ***P* < 0.01; ****P* < 0.001; n.s, not significant.


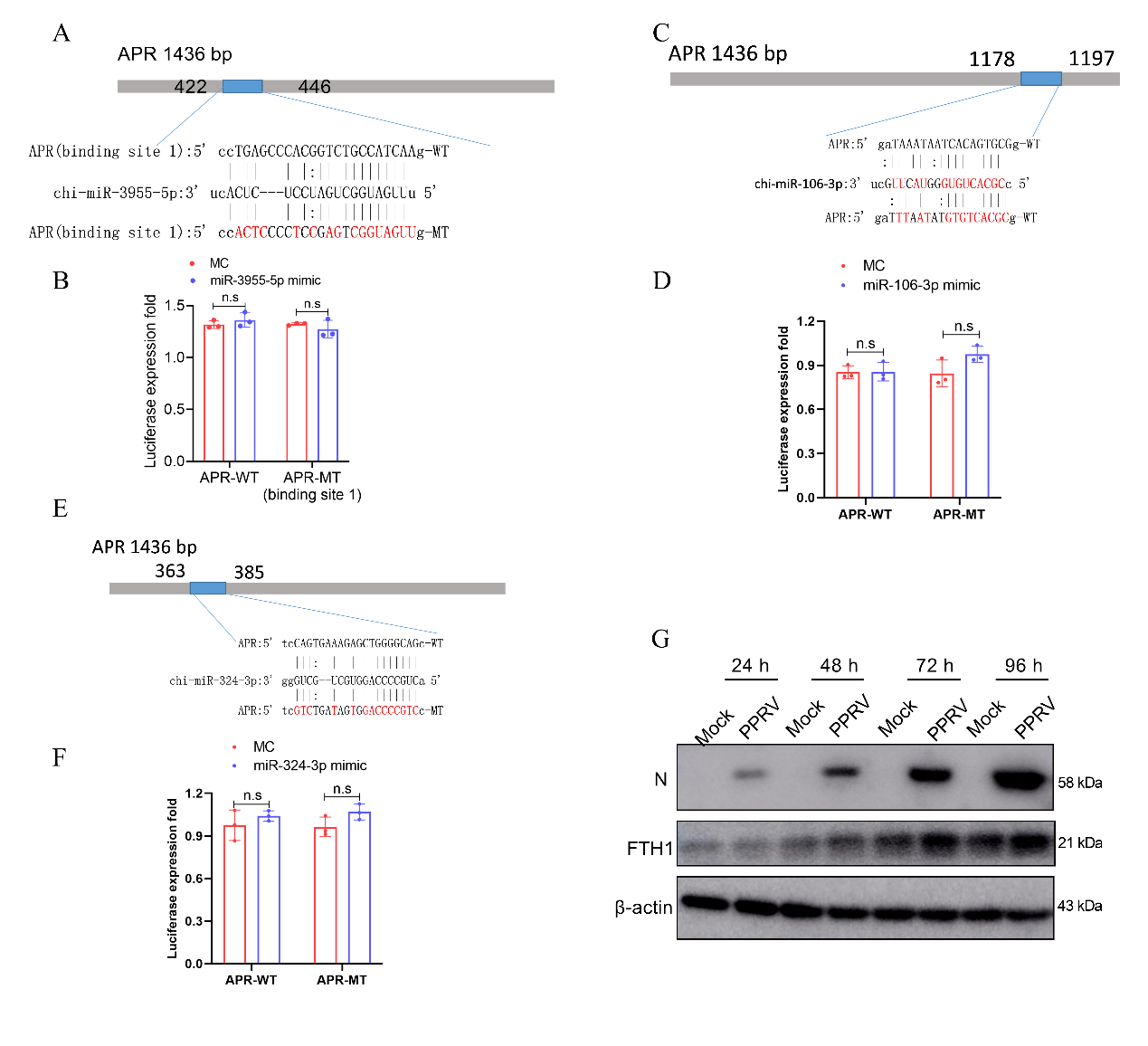


**Fig. S2.** Sequence alignment of (**A**) miR-3955-5p (binding site 1), (**C**) miR-106-3p and (**E**) miR-324-3p in APR, as predicated by TargetScan algorithm software. Dual-luciferase assay of HEK293T cells co-transfected with pmirGLO containing the putative miR-3955-5p binding site 1(**B**), miR-106-3p binding site (**D**) and miR-324-3p binding site(**F**), or pmirGLO containing the mutated binding sites, together with synthetic mature miR-3955-5p, miR-106-3p and miR-324-3p or mimic control (MC). (**G**) EECs or GFFs were infected with PPRV (MOI=3) for 48 h for the indicated times, and the protein levels PPRV N expression was measured by Western blotting. The data represent the mean ± SD of three independent experiments. *P* values were calculated using Student's *t* test. An asterisk indicates a comparison with the indicated control. **P* < 0.05; ***P* < 0.01; n.s., not significant.


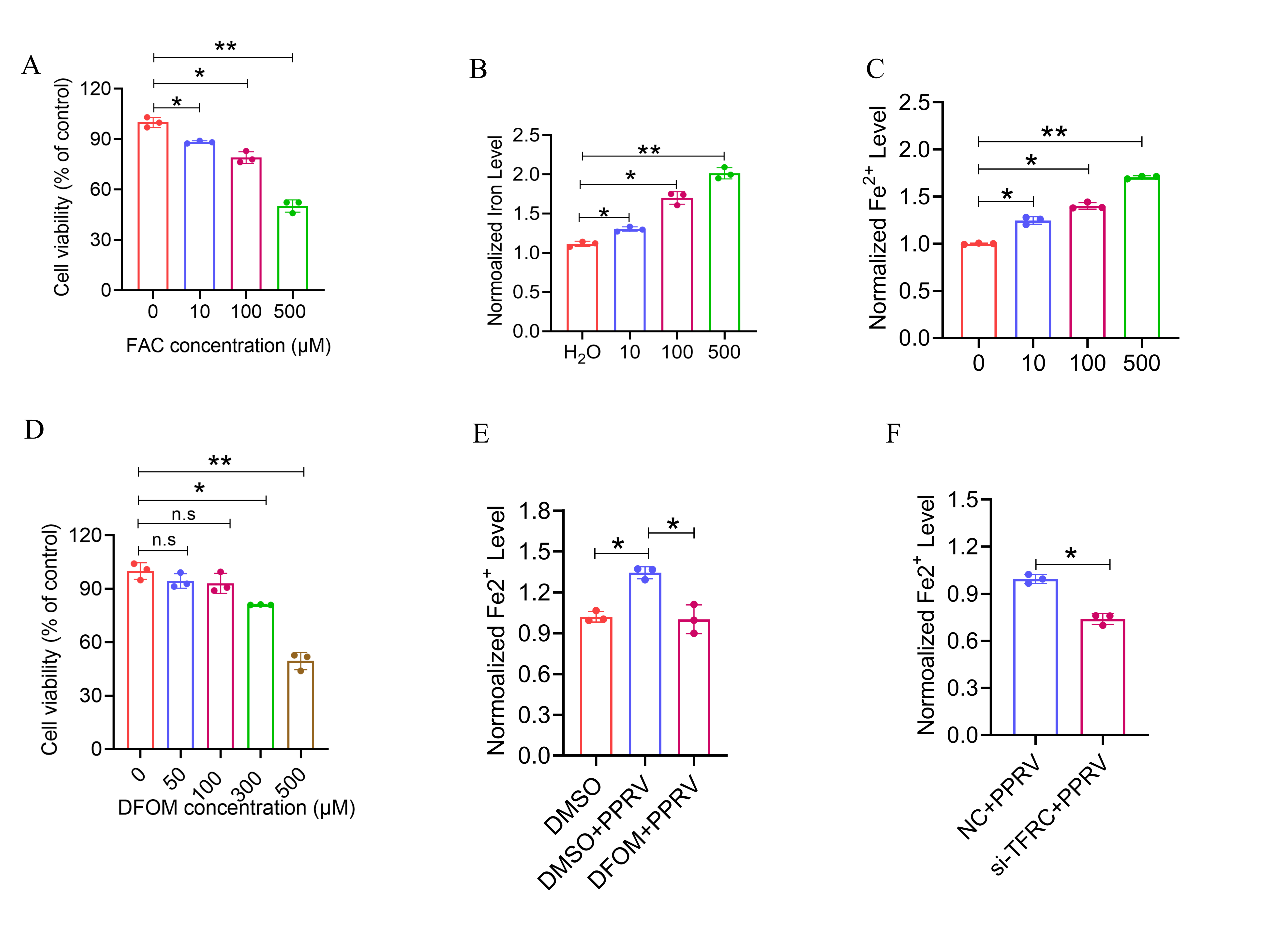


**Fig. S3.** EECs were treated with different concentrations of FAC for 48 h, and then cell viability (**A**) level of total iron (**B**) and Fe^2+^ (**C**) were detected. (**D** to **F**) EECs were treated with DFOM (100 μm) or mocked (DMSO) for 2 h, and then were infected by PPRV (MOI=3) for 48 h. Subsequently, cell viability (**D**), level of total iron (**E**) and Fe^2+^ (**F**) were detected. *P* values were calculated using Student's *t* test. An asterisk indicates a comparison with the indicated control. **P* < 0.05; ***P* < 0.01.


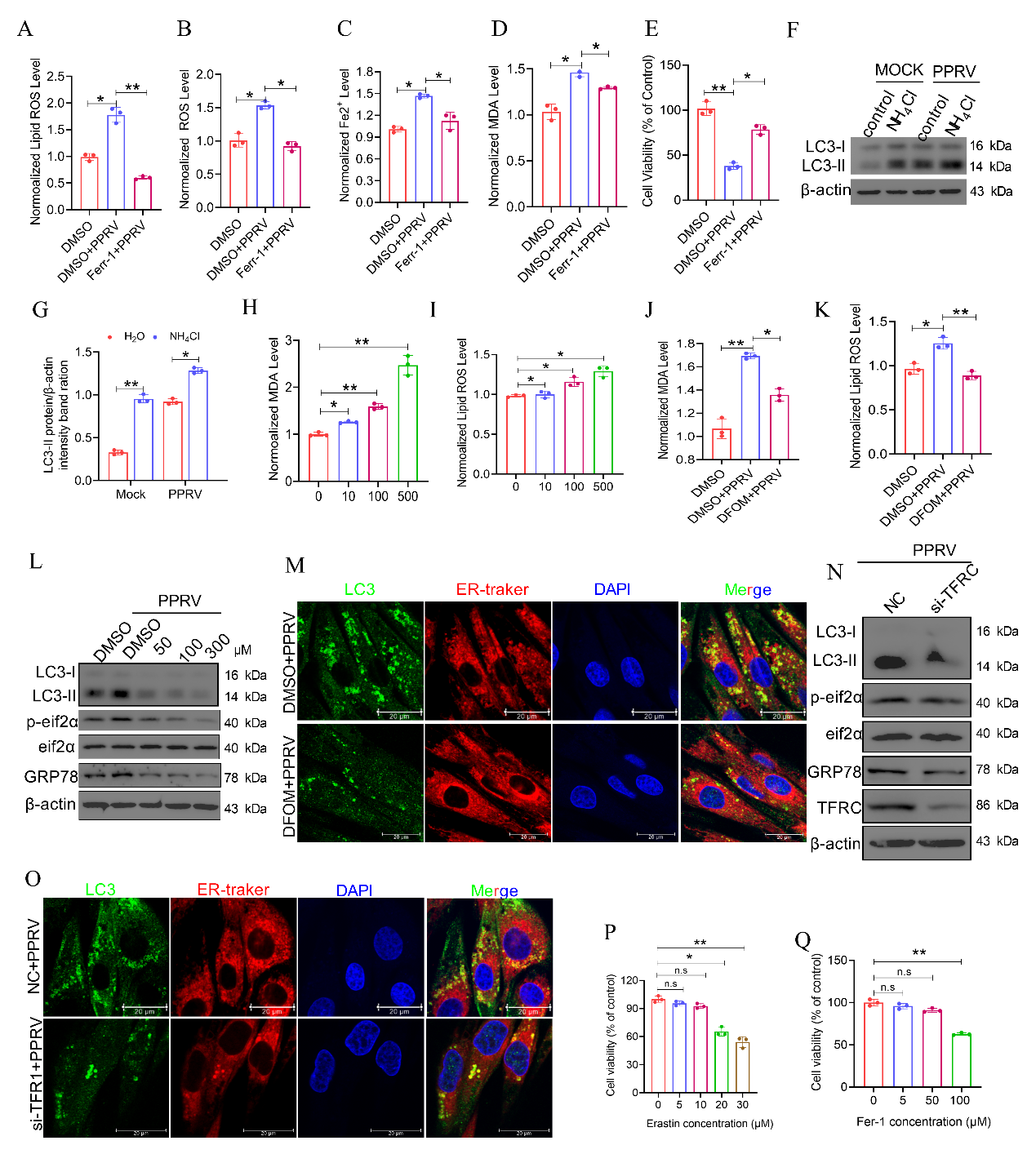


**Fig. S4** (**A** to **E**) EECs were treated with ferr-1 (50 μm) or mocked (DMSO) for 2 h, and then were infected by PPRV (MOI=3) for 48 h. Subsequently, (**A**) level of lipid ROS, (**B**) level of ROS, (**C**) level of Fe^2+^, (**D**) level of MDA were detected and (**E**) cell viability. (**F** and **G**) EECs with or without infection were treated with NH4Cl and then the conversion of LC3-I to LC3-II was assessed by Western blot. (**G**) The relative quantification of LC3 protein levels was determined by densitometry. (**H** and **I**) EECs were treated with different concentrations of FAC for 48 h, and then level of (**H**) MDA and (**I**) lipid ROS were detected. (**J** and **K**) EECs were treated with DFOM (100 μm) or mocked (DMSO) for 2 h, and then were infected by PPRV (MOI=3) for 48 h. Subsequently, level of (**J**) MDA and lipid (**K**) ROS were detected. (**L**) EECs were pre-treated with different concentrations of DFOM (100 μm) or mocked (DMSO) for 2 h, and then were infected by PPRV (MOI=3), 48 h later, the cells were subjected to Western blot for the analysis of the expression of LC3, phosphorylation of eIF2α, eIF2α and GRP78. (**M**) EECs were pre-treated with DFOM (100 μm) or mocked (DMSO) for 2 h, and then were infected by PPRV (MOI=3) for 48 h, subsequently, reticulophagy was determined by assessing the colocalization between LC3-positive autophagosomes (green) and ER Tracker labeled endoplasmic reticulum (red). (**N** and **O**) EECs were transfected with NC and si-TFRC for 24 h and then infected with PPRV at an MOI of 3, 48 h later, (**N**) the expression of LC3, phosphorylation of eIF2α, eIF2α and GRP78 were tested by Western blot and (**O**) reticulophagy was determined by assessing the colocalization between LC3-positive autophagosomes (green) and ER Tracker labeled endoplasmic reticulum (red). The cell nuclei were counterstained with DAPI. (**P** and **Q**) EECs were treated with different concentrations of erastin or Ferr-1for 48 h, and then cell viability was tested. β-actin was used as a loading control in Western blot analysis. The data represent the mean ± SD of three independent experiments. *P* values were calculated using Student's *t* test. An asterisk indicates a comparison with the indicated control. **P* < 0.05; ***P* < 0.01; ****P* < 0.001; n.s., not significant.


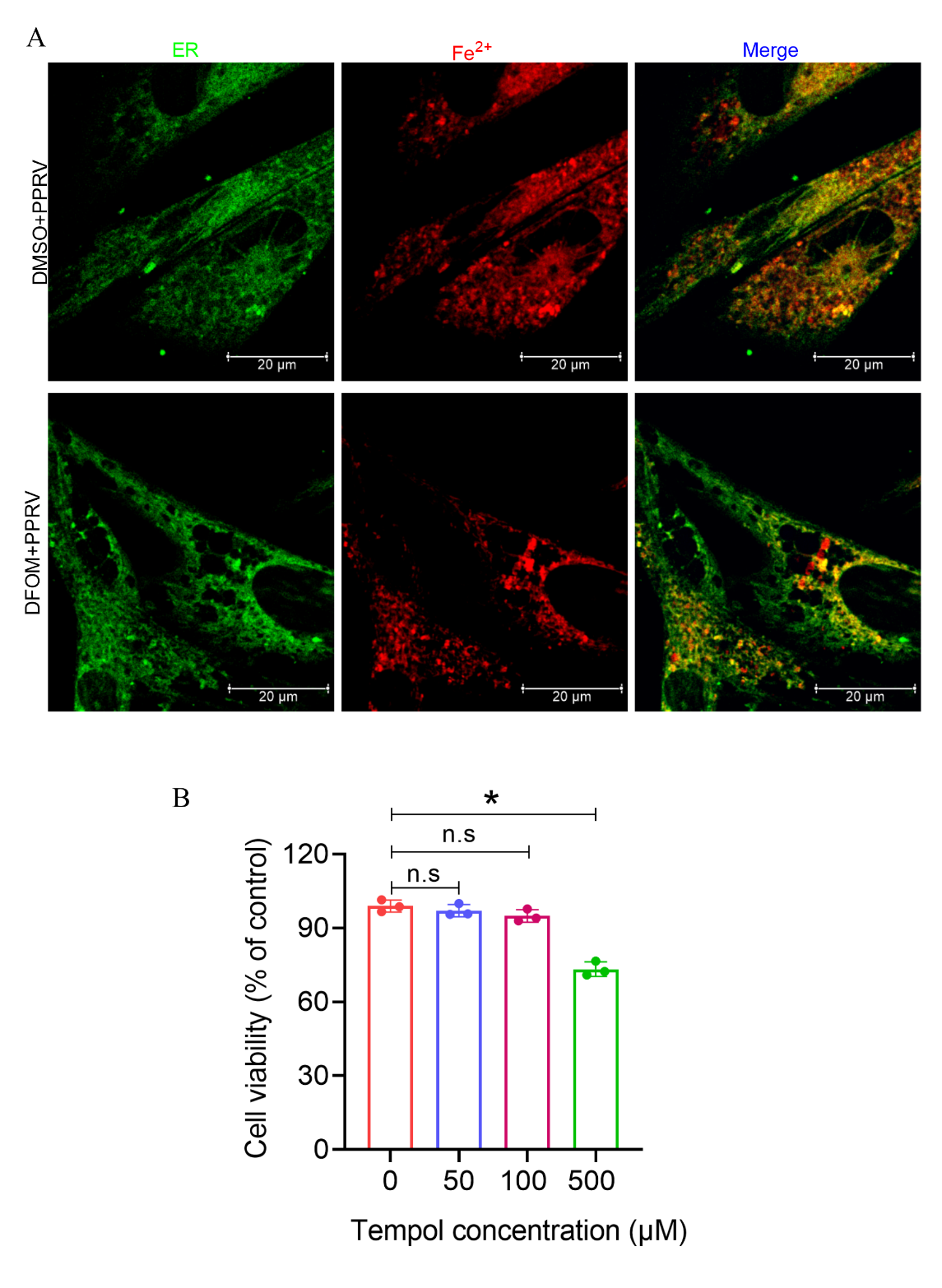


**Fig. S5** (**A**) EECs were were infected with PPRV at an MOI of 3 for 48 h, The colocalization between FerroOrange-stained intracellular iron (red) and ER Tracker labeled endoplasmic reticulum (green) was measured by confocal immunofluorescence microscopy. (**B**) The cell viability of EECs was measured by CCK-8 after treatment with different concentrations of Tempol for 48 h.


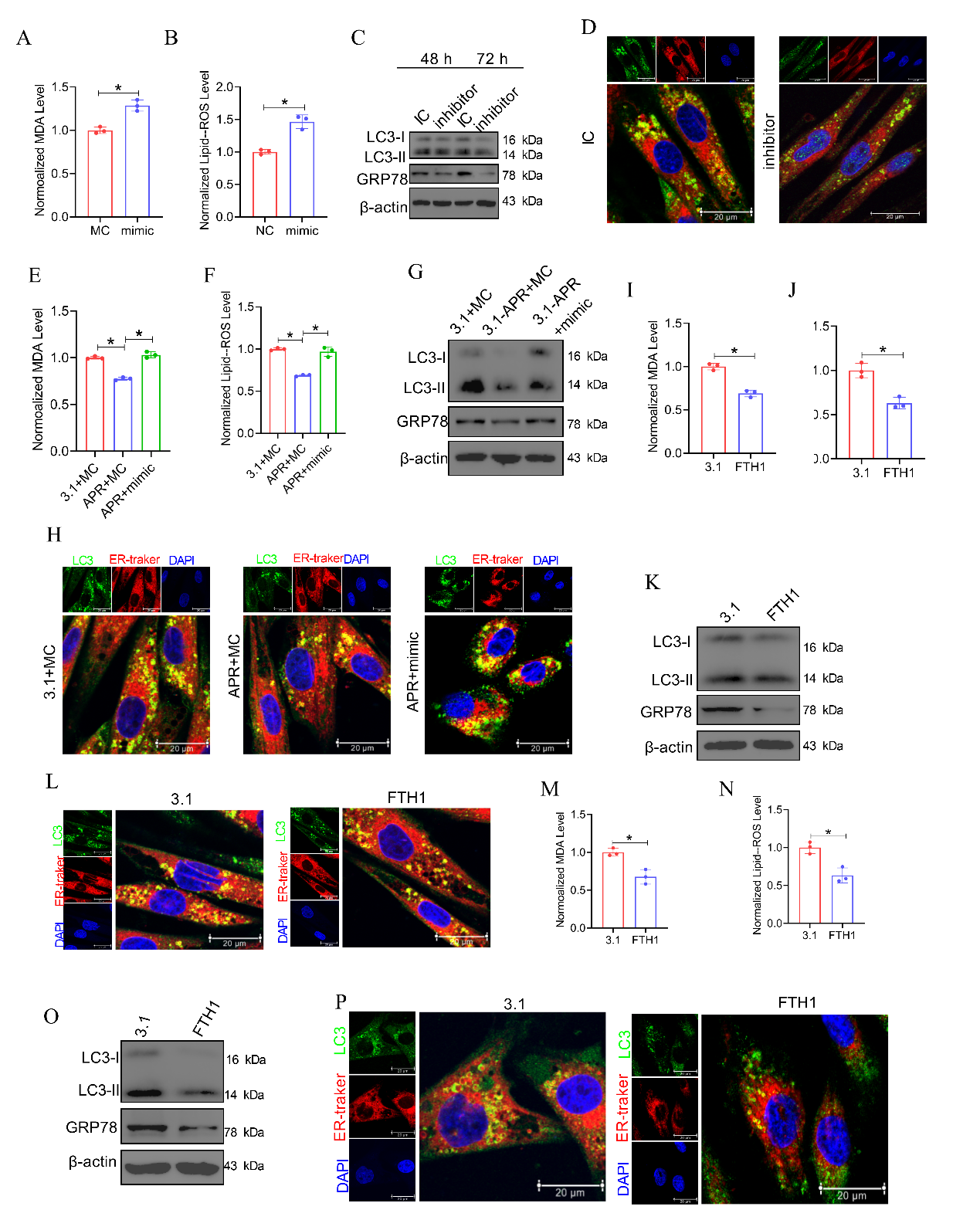


**Fig. S6** (**A** and **B**) EECs were transfected with control mimic (MC) or miR-3955-5p mimic for 24 h and then infected with PPRV at an MOI of 3 for 48 h, and then (**A**) level of MDA and (**B**) lipid ROS were detected. (**C** and **D**) EECs were transfected with IC or miR-3955-5p mimic for 24 h and then infected with PPRV at an MOI of 3. 48 h later, (**C**) the cells were subjected to Western blot for the analysis of the expression of the expression of LC3 and GRP78, and (**D**) reticulophagy was determined by assessing the colocalization between LC3-positive autophagosomes (green) and ER Tracker labeled endoplasmic reticulum (red). (**E** to **H**) EECs were cotransfected with pcDNA3.1 empty vector or pcDNA3.1-APR and control mimic (MC) or miR-3955-5p mimic for 24 h and then the cells were infected with PPRV at an MOI of 3. 48 h later, (**E**) level of MDA and (**F**) lipid ROS were detected, (**G**) the cells were subjected to Western blot for the analysis of the expression of the expression of LC3 and GRP78 and (**H**) reticulophagy was determined by assessing the colocalization between LC3-positive autophagosomes (green) and ER Tracker labeled endoplasmic reticulum (red). (**I** to **L**) EECs were transfected with pcDNA3.1 or pcDNA3.1-FTH1 for 24 h. 48 h later, (**I**) level of MDA and (**J**) lipid ROS were detected, (**K**) the cells were subjected to Western blot for the analysis of the expression of the expression of LC3 and (**L**) reticulophagy was determined by assessing the colocalization between LC3-positive autophagosomes (green) and ER Tracker labeled endoplasmic reticulum (red). (**M** to **P**) EECs were transfected with pcDNA3.1 or pcDNA3.1-FTH1 for 24 h and then the cells were treated with FAC (100 μM), (**M**) level of MDA and (**N**) lipid ROS were detected, (**O**) the cells were subjected to Western blot for the analysis of the expression of the expression of LC3 and GRP78 and (**P**) reticulophagy was determined by assessing the colocalization between LC3-positive autophagosomes (green) and ER Tracker labeled endoplasmic reticulum (red). The cell nuclei were counterstained with DAPI. β-actin was used as a loading control in Western blot analysis. An asterisk indicates a comparison with the indicated control. **P* < 0.05; ***P* < 0.01.
