# Supplementary material for: The long noncoding RNA APR attenuates PPRV infection-induced accumulation of intracellular iron to inhibit membrane lipid peroxidation and viral replication
Source: mBio. 2025 Mar 24;16(4):e00127-25. doi: 10.1128/mbio.00127-25 (PMC11980570; doi:10.1128/mbio.00127-25)
Supplement: Table S1 — Differential expression of lncRNAs in PPRV‑infected when compared mock‑infected EECs cells. [file mbio.00127-25-s0002.docx]

**Table S1** Differential expression of lncRNAs in PPRV‑infected when compared mock‑infected EECs cells

| Gene ID | Change fold |
| --- | --- |
| 106502218 | 5.950819 |
| 108635918 | 5.55227 |
| 108636242 | 4.414767 |
| 108637009 | 4.180301 |
| 108636281 | 4.047035 |
| 106502405 | 3.975482 |
| BGIG9925_30908 | 3.900193 |
| 108634954 | 3.79771 |
| 108635850 | 3.736695 |
| 108637526 | 3.55227 |
| 108636925 | 3.307851 |
| BGIG9925_31100 | 3.226313 |
| BGIG9925_32568 | 3.222121 |
| 108636557 | 3.199754 |
| 106502447 | 3.151732 |
| 108638258 | 2.900193 |
| BGIG9925_29876 | 2.900193 |
| 102185883 | 2.747703 |
| BGIG9925_29643 | 2.727789 |
| 108637780 | 2.677801 |
| 108633798 | 2.650834 |
| 108636127 | 2.592409 |
| 106503307 | 2.558879 |
| 108637734 | 2.502229 |
| 108637296 | 2.414767 |
| 108636124 | 2.39627 |
| BGIG9925_32973 | 2.376631 |
| 108636755 | 2.349178 |
| 108635056 | 2.324082 |
| 106502306 | 2.315231 |
| 108636393 | 2.262763 |
| 106502285 | 2.230342 |
| 102179243 | 2.20618 |
| 102191373 | 2.163228 |
| 102172314 | 2.151732 |
| 106503345 | 2.151732 |
| BGIG9925_30871 | 2.143465 |
| BGIG9925_29738 | 2.132367 |
| 102176410 | 2.092838 |
| 102188715 | 2.070118 |
| 108636637 | 2.059672 |
| 106502214 | 2.052196 |
| 102180551 | 2.047035 |
| 108634603 | 2.026051 |
| 108634529 | 1.999195 |
| 108638000 | 1.978667 |
| 102176916 | 1.940835 |
| 108634225 | 1.874838 |
| 102190882 | 1.845401 |
| 108633357 | 1.844159 |
| 108635557 | 1.84255 |
| 102169263 | 1.822191 |
| 102178647 | 1.815573 |
| 102179299 | 1.786983 |
| 102189982 | 1.775098 |
| 108636451 | 1.741704 |
| 106502882 | 1.73873 |
| 102187074 | 1.736695 |
| 102177684 | 1.718773 |
| 102178988 | 1.711748 |
| 102191471 | 1.70951 |
| BGIG9925_32950 | 1.707569 |
| 106502619 | 1.677801 |
| 108636385 | 1.647427 |
| 102175818 | 1.633407 |
| 102173615 | 1.626271 |
| 106502102 | 1.604738 |
| BGIG9925_29972 | 1.601852 |
| BGIG9925_29648 | 1.590338 |
| 108635095 | 1.568055 |
| 102172700 | 1.557003 |
| 108636397 | 1.547479 |
| 108635335 | 1.540015 |
| 106501764 | 1.528938 |
| 106503259 | 1.526492 |
| 102177673 | 1.525082 |
| BGIG9925_30845 | 1.509195 |
| 108636333 | 1.501644 |
| BGIG9925_30452 | 1.475253 |
| 108635298 | 1.473761 |
| 106503990 | 1.446475 |
| 106502039 | 1.433875 |
| 108635861 | 1.426664 |
| 102189941 | 1.423325 |
| 102171411 | 1.421164 |
| 102173353 | 1.414767 |
| 108634640 | 1.411619 |
| BGIG9925_30168 | 1.411056 |
| 108638502 | 1.409098 |
| 108638463 | 1.406947 |
| 108636751 | 1.389734 |
| BGIG9925_30879 | 1.382345 |
| 102183959 | 1.3513 |
| 108636407 | 1.345605 |
| 108634148 | 1.338595 |
| BGIG9925_33000 | 1.336141 |
| 106502121 | 1.325499 |
| 108636276 | 1.324164 |
| 102184696 | 1.315231 |
| 102179558 | 1.312375 |
| 102189102 | 1.306346 |
| 102172959 | 1.302292 |
| 108633927 | 1.289875 |
| 102168552 | 1.287204 |
| 108636752 | 1.281435 |
| 102173976 | 1.279924 |
| 108637659 | 1.248958 |
| 102168737 | 1.245813 |
| 106502027 | 1.245609 |
| 106502659 | 1.233701 |
| 106503007 | 1.220594 |
| 108636582 | 1.210296 |
| 106502445 | 1.205606 |
| 108633919 | 1.205048 |
| 102177353 | 1.190335 |
| 108636066 | 1.170841 |
| 106503509 | 1.168127 |
| 106502172 | 1.163228 |
| 102181471 | 1.158928 |
| 102184471 | 1.158753 |
| 102189342 | 1.152959 |
| 106503977 | 1.147573 |
| 102175195 | 1.142263 |
| 106502005 | 1.140144 |
| 102182335 | 1.12526 |
| BGIG9925_30451 | 1.125079 |
| 102168593 | 1.092838 |
| 106502045 | 1.085841 |
| 102181372 | 1.082497 |
| 102179832 | 1.079899 |
| 108635852 | 1.072277 |
| 108637776 | 1.063641 |
| 108638120 | 1.03739 |
| 102168946 | 1.013404 |
| 108637172 | 1.011815 |
| 106501937 | 1.010376 |
| 106502274 | 1.007722 |
| 102190274 | -1.01743 |
| 108637679 | -1.04236 |
| BGIG9925_30449 | -1.04898 |
| 106503278 | -1.07406 |
| 102169699 | -1.09027 |
| 102189648 | -1.22451 |
| BGIG9925_31561 | -1.25266 |
| 102179411 | -1.25766 |
| BGIG9925_31080 | -1.28413 |
| 106502936 | -1.31088 |
| BGIG9925_33027 | -1.32376 |
| BGIG9925_33002 | -1.34254 |
| 108637305 | -1.36214 |
| 102186229 | -1.36659 |
| 108638630 | -1.3683 |
| 108636067 | -1.48276 |
| 102185573 | -1.56555 |
| 108634978 | -1.56789 |
| 108637394 | -1.61765 |
| 102180967 | -1.66329 |
| 108635434 | -1.68925 |
| 102170571 | -1.72819 |
| 108637165 | -1.76131 |
| 108633238 | -1.76966 |
| 108638632 | -1.81405 |
| BGIG9925_31376 | -1.81405 |
| 106502883 | -1.92346 |
| 106503392 | -1.99462 |
| 106503029 | -2.2764 |
| 108636069 | -2.32337 |
| 108634157 | -2.36659 |
| 108636921 | -2.40193 |
| 102188008 | -2.52367 |
| 108637003 | -2.53652 |
| 102169208 | -2.55102 |
| BGIG9925_29762 | -2.64413 |
| 106502189 | -2.71452 |
| 106503672 | -2.79574 |
| BGIG9925_32056 | -2.80119 |
| BGIG9925_32563 | -2.88698 |
| 106502031 | -2.99233 |
| BGIG9925_29952 | -3.01137 |
| 108638305 | -3.07709 |
| BGIG9925_30838 | -3.18018 |
| BGIG9925_29676 | -3.36659 |
| 106501953 | -3.40359 |
| BGIG9925_30294 | -3.44882 |
| BGIG9925_31429 | -3.45736 |
| BGIG9925_29797 | -3.71452 |
| BGIG9925_31640 | -4.36659 |
| 106502872 | -4.6076 |
| BGIG9925_32398 | -4.99462 |
| BGIG9925_32868 | -5.55102 |
| BGIG9925_33028 | -6.3898 |
