# Supplementary material for: The long noncoding RNA APR attenuates PPRV infection-induced accumulation of intracellular iron to inhibit membrane lipid peroxidation and viral replication
Source: mBio. 2025 Mar 24;16(4):e00127-25. doi: 10.1128/mbio.00127-25 (PMC11980570; doi:10.1128/mbio.00127-25)
Supplement: Table S2 — lncRNA APR sequence. [file mbio.00127-25-s0003.docx]

**Table S2**. LncRNA APR sequence.

| AGCAGGAGCAGCATCGCGGCGCCGAGGAGCCGGGCGGCGGCGTTCGCGGCTCGGGCCATGGGTCTCAGCTCGGGCGGTCTGTGTCTGCGGCGGGAAGGCTGGCGGGAGCGCTGGCTGAGCGGGCTCTGTGGCTCCCCGAGCCGGGTGAACCCCTTTTATCCTGATCAGAAGGGAGTTGCCAGCCCGGGGGCCAGGGAAATTCCCTGTCCGGAAGGAAGGGGGCCCCGCCCGCCTCACGCGGAATCTGCGCACTCGGAGTGTCATCCCTGTACCCAAATCTCCACGCGACGCATTTGACCACTCAGCCTTTGAGTTTAGCCACTCTGGACATTTGACAAACCAGGACGGGCCAGAGTTTGTATTCCAGTGAAAGAGCTGGGGCAGCCAGAGAAATGAGTCCCAACCAAAGACCGGAGAAAGTCCTGAGCCCACGGTCTGCCATCAAGTCCAAAGAATACAGGCTTCTTCCATATACTCAGGTGATCTTGGAGACAGAGGATTCTGAATGATACTTATTTCAAAAGGTGGCTCTGCTACTGTATAATCTAGCTAGTTCATTGATTGACACTTCTGAAGTGGTTGACTGTATTAAAGAAATATGAGATCCAACTAGGTATGGTGGAAATTATGGTAAATGTAGAGAAAGCATGGCTTTGCTATATTTTAAATATGTGTATATTACATATATGTATGTTACATACGTTTATGTTATGCATCAAATGTGAATATCAGCAATCAATAATGTCCAATGAGCACATATTATAGCATCTGTGTGTTATTGAACCATGTAGATGGACAGATGAGGGGAACTCTCCACAGATTCTCTAACAGTTATAAATGCAACTAACAAAAAAATTTTATAAGTCATTTACTGGGCAAATGCATGAGTGTGTTATGATGTCTCTGGATAGTTTGGTGAAATCTCTGAGGTTAACAAAGTCAGGAGTTGGGAAGAGAGTCTTTAACATTCAGCCAACTCTCTACTGGCAAGGTGTCTTATCTAGCACCCAGCTGCACCATTATACAATAGTACTAACTGGGTTATTATTTCATCCATGTAGTAAATATCTGACAATCATTTATTTAGCACCAACACAGTCAGGCATTTTTCTGGAAGTGGGAATGAAGAGAGGTTAATGGACATGACCCTTACTCCTTAATCTACAAAAGGAAAACAGATAAATAATCACAGTGCGGTCCGGTTGTAATTCTAATGAGAGTGTACATAAATTGCTTTACTTCTTTTTGCCCAGCAGATCCTATCATCATCCCTAGAATTACCAGAAAAAGAAGACTCTTTCCCCAAACAGCTGATAAGTAAGAGCTAATTGTCCCCAGGCAGCTCATTTACCACCCTCCTTTCAAGTTTACTATCTCTCACCACCACACAGGCTAATAATACCATAATCTCTTTTTTATGTCTGCCTCAGGACCAT |
| --- |
