# Supplementary material for: The long noncoding RNA APR attenuates PPRV infection-induced accumulation of intracellular iron to inhibit membrane lipid peroxidation and viral replication
Source: mBio. 2025 Mar 24;16(4):e00127-25. doi: 10.1128/mbio.00127-25 (PMC11980570; doi:10.1128/mbio.00127-25)
Supplement: Table S3 — qRT-PCR primers used in this study. [file mbio.00127-25-s0004.docx]

**Table S3**. qRT-PCR primers used in this study.

| Target Gene | Forward primer (5’-3’) | Reverse primers (5’-3’) |
| --- | --- | --- |
| β-actin | CACGGTGCCCATCTACGA | CTTGATGTCACGGACGATTT |
| APR | TGGTAAATGTAGAGAAAGCATGGC | TGCTCATTGGACATTATTGATTGCT |
| FTH1 | CAACCTGGAGCTGTACGCC | TCTGCAGCTTCATCAGTCTCTC |
