# Supplementary material for: The long noncoding RNA APR attenuates PPRV infection-induced accumulation of intracellular iron to inhibit membrane lipid peroxidation and viral replication
Source: mBio. 2025 Mar 24;16(4):e00127-25. doi: 10.1128/mbio.00127-25 (PMC11980570; doi:10.1128/mbio.00127-25)
Supplement: Table S4 — Sequences of the examined miRNAs analyzed in this study. [file mbio.00127-25-s0005.docx]

**Supplementary Table 4.** Sequences of the examined miRNAs analyzed in this study.

| miRNA |  | Sequence (5’-3’) |
| --- | --- | --- |
| chi-miR-3955-5p |  | UUUGAUGGCUGAUCCUCUCACU |
| chi-miR-324-3p |  | ACUGCCCCAGGUGCUGCUGGG |
| chi-miR-106b-3p |  | CCGCACUGUGGGUACUUGCU |
